# Supplementary material for: Allosteric regulation and crystallographic fragment screening of SARS-CoV-2 NSP15 endoribonuclease
Source: Nucleic Acids Res. 2023 Apr 28;51(10):5255–70. doi: 10.1093/nar/gkad314 (PMC10250223; doi:10.1093/nar/gkad314)
Supplement: gkad314_Supplemental_Files [file gkad314_supplemental_files.zip › Supplementary datasets.zip!/183/Refine_4/01-BUSTER/Cycle-5/LIST.html]

Refining

**BUSTER 2.X**: A suite of programs for macromolecular structure refinement by maximum-likelihood and Bayesian methods  
Copyright © 1992-2020 Global Phasing Ltd.  
All rights reserved.  
Authors: G. Bricogne, E. Blanc, M. Brandl, C. Flensburg, P. Keller, W. Paciorek, P. Roversi, O. Smart, C. Vonrhein, T. Womack  

Please reference use of the software as:

- BUSTER-TNT 2.X, Global Phasing Ltd, Sheraton House, Cambridge CB3 0AX, UK

Version: 2.10.3 for linux64-ifort made on May 20 2020 at 01:14:34  
Licenced to: Diamond Light Source, Scientific Software  

```
---


### Parsing of the BUSTER cardfile

TITLE : autoBUSTER refinement

   Cell information

   Symmetry information

 Using CCP4 X-ray form factors.

   Missing structure chemical composition

 Using ATOM block for chemical composition.


   Shell information

### Parsing of the TNT cardfile


---


### Reading observations file

 Number of reflexions in input:  70521

      Number of accepted reflexions:  64732

      Number of rejected reflexions:      0

   Preparing complete.mtz containing:      70410 reflexions


   Reflexions in test set:            3243

   Reflexions in working set:        61489


---


### 1. Generating initial model

 2.10 * Shannon sampling rate. NX NY NZ:   288  288  240


  Effective Shannon rates are:    2.15    2.15    2.45


   Allocated BUFFER of size:     76.465 Mb.

   Initial Geometry Information


 No missing atoms declared


   Summary of model volumes

 No missing atoms declared: no prior computed


 Computing bulk solvent model scattering


 NCSMASK PDB radius for solvent mask:     2.15 Angstrom

 running PDBSET (setting cell and spacegroup P1) ... done
 running NCSMASK [1] (creating mask, SMOOTH 1.04563) ... done
 running MAPDUMP (to check if reduction to unit cell is needed) ... done
 running MAPMASK (converting mask to map) ... done

   Generation of babslv by theta-filter

   Electron density in frg mask:  0.420 Å-1

 FOBS  put on rough absolute scale by Wilson plot :
           K        =  0.53453E+02
           B        =     -4.815

 

### Cycle 0001: MaxLik scaling and refinement


   MaxLik Scaling Cycle 0001

   Wilson plot

   Statistics in resolution bins just after scaling
```

|  |  |
| --- | --- |
|  | - Rxpct factors plot- Rxpct factors table- <Log-Lik Gain>- Ln Sigma A- Correlation Coefficients |

```
   <Log-Likelihood> of null-hypothesis

Structure Factor Amplitudes


   Average Figure of Merit

 Switching off refinement of overall temperature factor


 Added B =    -1.4642 Å2 to all atoms in fragment


 Added B =    -1.4642 Å2 to B_SOLV

```
gelly refinement module
 Authors (2004-2009) O. S. Smart, C. Flensburg, W. Paciorek, C. Vonrhein, 
                     M. Brandl, T. O. Womack and G. Bricogne
         (2010-2011) O. S. Smart, C. Flensburg, W. Paciorek, C. Vonrhein, 
                     T. O. Womack and G. Bricogne
         (2012-2014) O. S. Smart, C. Flensburg, W. Paciorek, C. Vonrhein, 
                     and G. Bricogne
         (2015-2020) G. Bricogne, C. Flensburg, W. Paciorek, C. Vonrhein
 Copyright © 2004-2020 Global Phasing Ltd. 
 All rights reserved.
 gelly module compilation date May 20 2020 01:11:20
 
 gelly uses routines from TNT geometry. References: 
     Tronrud, D. E., Ten Eyck, L. F., Matthews, B. W. (1987)
     "An Efficient General-Purpose Least-Squares Refinement Program 
     for Macromolecular Structures". Acta Crystallogr A, 43:489-501.
     Tronrud, D. E. (1997). "The TNT Refinement Package", 
     in Macromolecular Crystallography, Part B, Eds Charlie Carter 
     and Robert Sweet, Methods in Enzymology, 277:306-319.
 wildcard matching function by Julian Robichaux http://www.nsftools.com

 No -random_seed flag found. Random number stream initialised using:
2147483562
2147483398

 Control flags set to:  -autoncs -hydrogens_input_occ2zero \
                     -hydrogens_output_occ2heavy -screen \
                     100 -screen_sigma \
                     3.0 -keeppdb \
                     1000000 -taomonitor_verbose \
                     2 
 

 ############### Starting setup via TNT routines

 Setting up tnt routines including license check:

                   TNT Stereochemistry Program
 Version: 5.22.3.0
 Licence for Diamond Light Source, Scientific Software is valid
 
 Using TNT geometry routines to load the tnt control file: gelly.tnt
 Using TNT routines to load initial coordinates from file fragment.cor
 Expecting 6125 atoms

 Found     2202 "NOTE BUSTER_RESIDUE_ATOM_DATA" cards
 Have copied information for    6125 atoms from TNT to gelly's store 
 
 The TNT WEIGHT cards used by this run:
 -----WEIGHT       BOND     2.0
 -----WEIGHT       ANGLE    2.0
 -----WEIGHT       IMPROPER 0.0
 -----WEIGHT       TORSION  2.0
 -----WEIGHT       PSEUDO   0.0
 -----WEIGHT       TRIGONAL 2.0
 -----WEIGHT       PLANE    5.0
 -----WEIGHT       CONTACT  5.0
 -----WEIGHT       BCORREL  10.0
 -----WEIGHT       CHIRAL   5.0
 -----WEIGHT       NCS      50.0
 -----WEIGHT       IDEAL    4.0
 -----WEIGHT       RFACTOR  7.37
 
 Dictionary versions picked up from NOTE BUSTER_RESTRAINT_DICT cards as: 
 ------ protgeo_eh99.dat (V1.6.4.17) 20191001  STANDARD AMINO ACID DICTIONARY. BONDS AND ANGLES FROM ENGH AND HUBER EH99. OTHER VALUES BASED ON PREVIOUS TNT OR TAKEN FROM CCP4. INCLUDES HYDROGEN ATOMS.
 ------ exoticaa.dat (V1.3.4.15) 20200327  COLLECTION OF NON-STANDARD AMINO ACIDS, MAINLY EH91 WITHOUT IDEAL DISTANCE INFO
 ------ nuclgeo.dat (V1.13.4.8) 20190909
 ------ bcorrel.dat (V1.15.10.2) 20190616
 ------ contact.dat (V1.15.12.10) 20191121
 ------ idealdist_contact.dat (V1.3.4.12) 20191001  IDEAL-DISTANCE CONTACT TERM DATA AS USED IN PROLSQ. VALUES USED HERE ARE BASED ON DATA HARVESTING OF PDB AS OF 20190109.
 ------ restraints for LIG (Unknown) from cif dictionary Z2856434903.cif
 ------ restraints for CIT (CITRIC ACID) from cif dictionary CIT.cif; generated by GRADE_PDB_LIGAND 1.2.12 (October 4 2016) using MOGUL 1.7.2(RC1), CSD as538be, with quantum mechanics RM1 ((DatabaseID PDB-CIT))
 ------ assume.dat (V1.9.4.2) 20180705
 
 Have picked up spacegroup name for pdb output as 'P 63      '
 Setting up Chain/Residue arrays
 
 Have command line specification -autoncs*
     Have found       1 NCS relations
     Pairwise matching residue number and type between chains: 
                    A        B        S    
          A         .       99%       -   
          B                  .        -   
          S                           .   
       Table key--                                                       
          all   : two chains have 100% match and same number of residues 
         multi  : ditto and they contribute to a multi chain ncs cluster 
          ***%  : two chains have >80% matching residue number/type      
           -    : chains appear to be unrelated                          
      chain A     have identified ncs to     1 other chains. So will weight each autoncs by (       6.666/#)=      6.6660
      chain B     have identified ncs to     1 other chains. So will weight each autoncs by (       6.666/#)=      6.6660
      chain S     have identified ncs to     0 other chains. 
     For .Gelly cards produced see below - after pruning
 
 Setting up BUSTER_SET groups
 Have found    89 BUSTER_SET cards
 Now silently applying automatic/dictionary set definitions. For a verbose listing use '-verbose_set' flag.
 Applying user defined set definitions:
 =====NOTE BUSTER_SET ncsautoXcld = Water --> set 'ncsautoXcld' now has   581 atoms 
 =====NOTE BUSTER_SET ncsautoABset = Chain_B \ ncsautoXcld --> set 'ncsautoABset' now has  2774 atoms 
 Have defined    48 sets: 
      set:                 'All' has   6125 atoms in   1281 residues - automatically defined
      set:               'Empty' has      0 atoms in      0 residues - automatically defined
      set:            'ExoticAA' has      0 atoms in      0 residues - defined by dictionary file
      set:           'ExoticNUC' has      0 atoms in      0 residues - defined by dictionary file
      set:              'NotHET' has   5514 atoms in    698 residues - defined by dictionary file
      set:             'Chain_A' has   2770 atoms in    350 residues - automatically defined
      set:             'Chain_B' has   2774 atoms in    350 residues - automatically defined
      set:             'Chain_S' has    581 atoms in    581 residues - automatically defined
      set:               'Water' has    581 atoms in    581 residues - defined by dictionary file
      set:          'StdProtein' has   5514 atoms in    698 residues - defined by dictionary file
      set:             'Protein' has   5514 atoms in    698 residues - defined by dictionary file
      set:                'Back' has   2794 atoms in    698 residues - defined by dictionary file
      set:                'Side' has   2720 atoms in    650 residues - defined by dictionary file
      set:        'RnaCanonical' has      0 atoms in      0 residues - defined by dictionary file
      set:        'DnaCanonical' has      0 atoms in      0 residues - defined by dictionary file
      set:     'RnaDnaCanonical' has      0 atoms in      0 residues - defined by dictionary file
      set:     'DnaRnaCanonical' has      0 atoms in      0 residues - defined by dictionary file
      set:         'RnaExtended' has      0 atoms in      0 residues - defined by dictionary file
      set:         'DnaExtended' has      0 atoms in      0 residues - defined by dictionary file
      set:      'RnaDnaExtended' has      0 atoms in      0 residues - defined by dictionary file
      set:      'DnaRnaExtended' has      0 atoms in      0 residues - defined by dictionary file
      set:                 'Dna' has      0 atoms in      0 residues - defined by dictionary file
      set:                 'Rna' has      0 atoms in      0 residues - defined by dictionary file
      set:              'RnaDna' has      0 atoms in      0 residues - defined by dictionary file
      set:              'DnaRna' has      0 atoms in      0 residues - defined by dictionary file
      set:             'Nucleic' has      0 atoms in      0 residues - defined by dictionary file
      set:             'Polymer' has   5514 atoms in    698 residues - defined by dictionary file
      set: 'BufferWeichenberger' has     13 atoms in      1 residues - defined by dictionary file
      set:    'BufferAdditional' has      0 atoms in      0 residues - defined by dictionary file
      set:              'Buffer' has     13 atoms in      1 residues - defined by dictionary file
      set:         'MetalAlkali' has      0 atoms in      0 residues - defined by dictionary file
      set:  'MetalAlkalineEarth' has      0 atoms in      0 residues - defined by dictionary file
      set:     'MetalTransition' has      0 atoms in      0 residues - defined by dictionary file
      set: 'MetalPostTransition' has      0 atoms in      0 residues - defined by dictionary file
      set:    'MetalLanthanides' has      0 atoms in      0 residues - defined by dictionary file
      set:               'Metal' has      0 atoms in      0 residues - defined by dictionary file
      set:         'SugarAlphaD' has      0 atoms in      0 residues - defined by dictionary file
      set:         'SugarAlphaL' has      0 atoms in      0 residues - defined by dictionary file
      set:               'Sugar' has      0 atoms in      0 residues - defined by dictionary file
      set:               'Other' has     30 atoms in      2 residues - defined by dictionary file
      set:            'Hydrogen' has      0 atoms in      0 residues - defined by dictionary file
      set:         'nonHydrogen' has   6125 atoms in   1281 residues - defined by dictionary file
      set:             'OccZero' has      0 atoms in      0 residues - defined by dictionary file
      set:          'OccNonZero' has   6125 atoms in   1281 residues - defined by dictionary file
      set:             'OccFull' has   6076 atoms in   1278 residues - defined by dictionary file
      set:          'OccNonFull' has     49 atoms in      3 residues - defined by dictionary file
      set:         'ncsautoXcld' has    581 atoms in    581 residues - user specified
      set:        'ncsautoABset' has   2774 atoms in    350 residues - user specified
 No command line arguments '-occ_allhet' or '-occ_restyp' so will not setup automated occcupancy refinement
 
 Interpreting CONSTANT and FREE cards: 
 =====CONSTANT     OCC
 Have found and interpreted      1 CONSTANT cards in the TNT input. 
 Before any COMBINE - Number of atoms with fixed XYZ =     0
 Before any COMBINE - Number of atoms with fixed B   =     0
 Before any COMBINE - Number of atoms with fixed OCC =  6125
 Before any COMBINE - Number of atoms with fixed BIJ =   581
                            cf total number of atoms =  6125
 
 There are no COMBINE cards to be found in the TNT card input 
 
 There are no NOTE BUSTER_COMBINE_RES cards to be found in the TNT card input 

 ############### Working out full connectivity list


 ############### Working out equivalent atoms


 Residue with most atoms given below:
        B|401:C01(LIG)      
        B|401:N02(LIG)      
        B|401:C03(LIG)      
        B|401:C04(LIG)      
        B|401:N05(LIG)      
        B|401:C06(LIG)      
        B|401:C07(LIG)      
        B|401:C08(LIG)      
        B|401:C09(LIG)      
        B|401:C10(LIG)      
        B|401:C11(LIG)      
        B|401:N12(LIG)      
        B|401:C13(LIG)      
        B|401:O14(LIG)      
        B|401:C15(LIG)      
        B|401:C16(LIG)      
        B|401:C17(LIG)      


 Have no '-equiv_check_plus' argument - so will take atom type into
 account when checking for equivalence of otherwise identical atoms.
 

 ############### Looking at dihedrals


 ############### Looking at chirals

 NOTE BUSTER_CHIRAL_ANGLECUT card found - read value=    -31.00 degs from it
 NOTE BUSTER_CHIRAL_SIGMA card found - read value=      1.70 degs from it
 RANGE allowed for B variables in refinement:   from      3.00000 to    300.00000

 ############### Preparing for PDB output


 ############### Processing extra restraints explicitly defined

 No 'NOTE BUSTER_TARGET' cards or -target?? args found (used to read coordinates files for soft ncs to external)
 No 'NOTE BUSTER_OCCSUM'     cards found - so not using sum of occupancy restraints
 No 'NOTE BUSTER_DISTANCE'   cards found - so not setting up any additional atom-atom distance restraints
 No 'NOTE BUSTER_UTILANGLE'  cards found - so not setting up any additional restraints on any angle
 No 'NOTE BUSTER_UTILTOR'    cards found - so not setting up any additional sinusoidal restraints on torsions
 
 --- Have some similarity definitions 
     Similarity restraints on RMSD use superposition routines from: 
     Coutsias, E.A., Seok, C., Dill, K.A.(2004)
     "Using quaternions to calculate RMSD", J. Comput. Chem., 25:1849-1857 

 Have found    1 'NOTE BUSTER_SIM_DEFINE' cards 
     Maximum number of chains involved in any SIM group (excluding the template)=     1


 ########## Interpreting card: NOTE BUSTER_SIM_DEFINE ncsautoAB ncsautoABset A
     Number of atoms in template set=  2774
     Number of related chains (other than template) in SIM set=    1
     List of chains to be restrained (excluding template)=   A   
     Pairwise RMSD, XYZ in Angstroms, for SIM group: ncsautoAB
                 B         A   
     B         -----     ----- 
     A         0.595     ----- 
     Overall RMSD,XYZ for the SIM group is     0.595 Angstroms
     Pairwise rms deviation of [individual B's minus their set/chain average] in Angs**2
                 B         A         Bavg
     B         -----     -----     56.64
     A         14.86     -----     69.22
     Overall RMSD,B   for the SIM group is    14.858 Angs**2
     The number of common atoms for the above fits:
                 B         A   
     B         -----     ----- 
     A          2757     ----- 
     (compared to the template set that has    2774 atoms in it)
     List of atoms that are absent in one or more chains for this group: 
                                    B    A   
                    C01     401     +    -
                    N02     401     +    -
                    C03     401     +    -
                    C04     401     +    -
                    N05     401     +    -
                    C06     401     +    -
                    C07     401     +    -
                    C08     401     +    -
                    C09     401     +    -
                    C10     401     +    -
                    C11     401     +    -
                    N12     401     +    -
                    C13     401     +    -
                    O14     401     +    -
                    C15     401     +    -
                    C16     401     +    -
                    C17     401     +    -
     (key  +  atom present for this chain 
           -  atom absent  for this chain)
 transforms for each of the superpositions orthogonal matrix r11 r12 r13 r21 r22 r23 r31 r32 r33 tx ty tz (PDBSET syntax): 
TRANS_BA -0.877836 -0.478857  0.009973 -0.478798  0.877890  0.007840 -0.012509  0.002108 -0.999920    45.351   -12.796    15.707
 reverse transforms 
TRANS_AB -0.877836 -0.478798 -0.012509 -0.478857  0.877890  0.002108  0.009973  0.007840 -0.999920    33.881    32.917    15.354
 
 Setting up LSSR interatomic distance restraints
 Interpreting card: NOTE BUSTER_SIM_RESTRAIN_LSSR ncsautoAB 6.666
        setting up interatomic distance pair restraints with:
        Dmin=         0.00
        Dmax=         5.50
        sigma=        0.20
        Vmax=         3.00
        weight=       6.67
        number of distance pairs defined for this set is      34331
 Have not supplied either '-sim_swap_equiv' or '-sim_swap_equiv_plus' argument 
   so will not try swaping equivalent atoms to improve NCS and/or target agreement.
 
 NCS by LSSR outlier analysis
 -autoncs option automatic NCS prune routine -------- 
 Residues pruned out by gradient condition:
           A|54     A|131     A|132     A|135     A|145     A|147     A|148
          A|149     A|150     A|151     A|173     A|179     A|182     A|187
          A|188     A|189     A|215     A|216     A|217     A|219     A|220
          A|231     A|234     A|235     A|238     A|239     A|241     A|242
          A|244     A|246     A|253     A|259     A|260     A|264     A|288
          A|339     A|340     A|344     A|345      B|54     B|131     B|132
          B|135     B|145     B|147     B|148     B|149     B|150     B|151
          B|173     B|179     B|182     B|187     B|188     B|189     B|215
          B|216     B|217     B|219     B|220     B|231     B|234     B|235
          B|238     B|239     B|241     B|242     B|244     B|246     B|253
          B|260     B|264     B|288     B|339     B|340     B|344     B|345
       
 Residues pruned out by function/max condition:
          A|149!    A|187!    A|220!   A|ENDA     B|149!    B|187!    B|220!
         B|ENDA 
  
 Total number of residues for which LSSR restraints have been pruned out is       79 out of      698 participating
 Number of LSSR restraints pruned out is      5276 this is   15.4% of the total           34331
  
 -autoncs .Gelly cards suitable for adaptation for manual definition/pruning
           (note auto pruning is done in a slightly different way so exact results may differ slightly)
 -autoncs option .Gelly cards also written to file  auto_gelly_cards.txt
NOTE BUSTER_SET ncsautoXcld = Water    # card added - produced by -autoncs
NOTE BUSTER_SET ncsautoXcld = ncsautoXcld + { B|54 B|131 B|132 B|135 B|145 }    # card added - produced by -autoncs
NOTE BUSTER_SET ncsautoXcld = ncsautoXcld + { B|147 B|148 B|149 B|150 B|151 }    # card added - produced by -autoncs
NOTE BUSTER_SET ncsautoXcld = ncsautoXcld + { B|173 B|179 B|182 B|187 B|188 }    # card added - produced by -autoncs
NOTE BUSTER_SET ncsautoXcld = ncsautoXcld + { B|189 B|215 B|216 B|217 B|219 }    # card added - produced by -autoncs
NOTE BUSTER_SET ncsautoXcld = ncsautoXcld + { B|220 B|231 B|234 B|235 B|238 }    # card added - produced by -autoncs
NOTE BUSTER_SET ncsautoXcld = ncsautoXcld + { B|239 B|241 B|242 B|244 B|246 }    # card added - produced by -autoncs
NOTE BUSTER_SET ncsautoXcld = ncsautoXcld + { B|253 B|260 B|264 B|288 B|339 }    # card added - produced by -autoncs
NOTE BUSTER_SET ncsautoXcld = ncsautoXcld + { B|340 B|344 B|345 B|ENDA }    # card added - produced by -autoncs
NOTE BUSTER_SET ncsautoABset = Chain_B \ ncsautoXcld    # card added - produced by -autoncs
NOTE BUSTER_SIM_DEFINE ncsautoAB ncsautoABset A    # card added - produced by -autoncs
NOTE BUSTER_SIM_RESTRAIN_LSSR ncsautoAB 6.666    # card added - produced by -autoncs

 LSSR function contribution before pruning     32067.6
                             after pruning      9343.5
 Graph of which residues pruned from LSSR: ncs_pruned.mtv
 Graph of LSSR function outliers ncs_function_outliers.mtv
 Graph of LSSR gradient outliers ncs_gradient_outliers.mtv
 LSSR - analysis of distance difference distribution for NCS
         DeltaD less than 0.5 Angs:  proportion of population= 98.81% average DeltaD of subset     0.012 Angs
 Histogram of LSSR deltaD distribution ncs_histogram.mtv
 
 Interpreting card: NOTE BUSTER_TLS_SET tls1 { A|* }
 Interpreting card: NOTE BUSTER_TLS_SET tls2 { B|* }
 Interpreting card: NOTE BUSTER_TLS_SET tls1 { A|* }
 Interpreting card: NOTE BUSTER_TLS_T   tls1     -2.09805E-01 -1.15711E-01  3.73362E-01 -2.40920E-02  4.96139E-02  2.21940E-02
 Interpreting card: NOTE BUSTER_TLS_L   tls1      1.24514E+00  1.05226E+00  6.66191E-01  1.62361E-01 -3.88521E-01  3.54587E-01
 Interpreting card: NOTE BUSTER_TLS_S   tls1      2.06506E-01 -1.53727E-01  1.93700E-01 -1.05534E-01 -6.67227E-02 -9.37109E-02 -4.76199E-02 -4.26923E-02
 Interpreting card: NOTE BUSTER_TLS_O   tls1     -2.33748E+01 -8.36624E+01  1.14308E+01
 Interpreting card: NOTE BUSTER_TLS_FIX tls1 ALL
 Interpreting card: NOTE BUSTER_TLS_SET tls2 { B|* }
 Interpreting card: NOTE BUSTER_TLS_T   tls2     -2.19975E-01 -1.58351E-01  4.94077E-01  1.93068E-02  4.62273E-02  2.44113E-03
 Interpreting card: NOTE BUSTER_TLS_L   tls2      1.30038E+00  8.42752E-01  9.70856E-01  6.65317E-01 -8.38129E-01 -5.84650E-01
 Interpreting card: NOTE BUSTER_TLS_S   tls2     -2.69202E-02  1.09150E-01 -8.14403E-02  6.77598E-02  1.92600E-01  1.97964E-02 -6.29584E-02  2.14661E-02
 Interpreting card: NOTE BUSTER_TLS_O   tls2      2.20570E+01 -7.19562E+01  3.49479E+00
 Interpreting card: NOTE BUSTER_TLS_FIX tls2 ALL


 TLS information. Total number of groups:     2


 Group   Natoms    FixRB   FixTLS   Group Tag    Group Spec
     1     2770      Y        Y     tls1    { A|* }
     2     2774      Y        Y     tls2    { B|* }
 
NOTE BUSTER_TLS_SET tls1 { A|* }
NOTE BUSTER_TLS_T   tls1     -2.09805E-01 -1.15711E-01  3.73362E-01 -2.40920E-02  4.96139E-02  2.21940E-02
NOTE BUSTER_TLS_L   tls1      1.24514E+00  1.05226E+00  6.66191E-01  1.62361E-01 -3.88521E-01  3.54587E-01
NOTE BUSTER_TLS_S   tls1      2.06506E-01 -1.53727E-01  1.93700E-01 -1.05534E-01 -6.67227E-02 -9.37109E-02 -4.76199E-02 -4.26923E-02
NOTE BUSTER_TLS_O   tls1     -2.33748E+01 -8.36624E+01  1.14308E+01
NOTE BUSTER_TLS_FIX tls1 ALL
 
NOTE BUSTER_TLS_SET tls2 { B|* }
NOTE BUSTER_TLS_T   tls2     -2.19975E-01 -1.58351E-01  4.94077E-01  1.93068E-02  4.62273E-02  2.44113E-03
NOTE BUSTER_TLS_L   tls2      1.30038E+00  8.42752E-01  9.70856E-01  6.65317E-01 -8.38129E-01 -5.84650E-01
NOTE BUSTER_TLS_S   tls2     -2.69202E-02  1.09150E-01 -8.14403E-02  6.77598E-02  1.92600E-01  1.97964E-02 -6.29584E-02  2.14661E-02
NOTE BUSTER_TLS_O   tls2      2.20570E+01 -7.19562E+01  3.49479E+00
NOTE BUSTER_TLS_FIX tls2 ALL


 TLS: mmCIF group specification begin


#
loop_
_pdbx_refine_tls_group.id
_pdbx_refine_tls_group.refine_tls_id
_pdbx_refine_tls_group.beg_PDB_ins_code
_pdbx_refine_tls_group.beg_auth_asym_id
_pdbx_refine_tls_group.beg_auth_seq_id
_pdbx_refine_tls_group.end_PDB_ins_code
_pdbx_refine_tls_group.end_auth_asym_id
_pdbx_refine_tls_group.end_auth_seq_id
_pdbx_refine_tls_group.selection
_pdbx_refine_tls_group.selection_details
   1   1 . A   -1 . A  346 . '{ A|* }'
   2   1 . A  401 . A  401 . '{ A|* }'
   3   2 . B   -1 . B  346 . '{ B|* }'
   4   2 . B  401 . B  401 . '{ B|* }'
#


 TLS: mmCIF group specification end


 Interpreting card: NOTE BUSTER_ANISO_SET Empty
 Interpreting card: NOTE BUSTER_ANISO_WEIGHT SCA  16.0
 Interpreting card: NOTE BUSTER_ANISO_WEIGHT SPH   0.6    0.6
 Interpreting card: NOTE BUSTER_ANISO_WEIGHT SIM   7.8    4.8    2.6    0
 Interpreting card: NOTE BUSTER_ANISO_WEIGHT RIG  21.5   21.5    3.7    0


 After TLS and ANISO: Number of atoms with fixed/combined xyz =     0
 After TLS and ANISO: Number of atoms with fixed/combined B   =     0
 After TLS and ANISO: Number of atoms with fixed/combined OCC =  6125
 After TLS and ANISO: Number of atoms with fixed/combined BIJ =  6125
 After TLS and ANISO: Number of atoms in a TLS group          =  5544
                                     cf total number of atoms =  6125
 

 ############### Setup state vector

 No free_vect_ulim_xyz argument found: using default of  100.000
 No free_vect_llim_xyz argument found: using default of -100.000
 Number of parameters added for TLS description                 =       0
 Number of parameters to describe molecular conformation        =   24500

 ############### Setup free vector

 Number of free parameters to be adjusted in refinement   =   24500

 ############### Setting up own geometry


 No command line argument '-tnt_fn' so will use gelly to do geometry function calculations

 ============= Call TNT functions


 ============= Get TNT symmetry information

 
      Getting symmetry operators from TNT. 
          gelly will classify symmetry using pdb-like convention: 
               SYMOP   SYMMETRY
              NNNMMM   OPERATOR
                1555   X,Y,Z
                2555   -Y,X-Y,Z
                3555   -X+Y,-X,Z
                4555   -X,-Y,1/2+Z
                5555   Y,-X+Y,1/2+Z
                6555   X-Y,X,1/2+Z
          where NNN -> operator number and MMM -> translation vector
 

 ============= Own Gelly geometry/restraints setup


 ----------- Identify bond-types


    Setting up GEOMBONDREC_TYPE_HASH* to be able to map a given bond type (e.g.
    "single") to an internal integer (GBOND_TYPE=1) etc.

    Using default  GEOMBONDREC_TYPE_HASH_STR='SING* 1 DOUB* 2 TRIP* 3 AROM* 4 DELO* 4 METAL* 5':
    to override default use a NOTE BUSTER_GEOMBONDREC_TYPE_HASH_STR gelly card.

 ----------- Classify torsion into peptide omega and other


 ----------- Setup of non-bonded terms

 Ideal-distance contact term setup:
    Restraint function constants (normally these values are from NOTE BUSTER_IDEAL_CONTACT_* cards)
    Sigma for general contacts      0.200 Angs
    Sigma for 1-4 contacts          0.100 Angs (contacts across a torsion)
    Sigma for 1-5 contacts          0.150 Angs
    Sigma for 1-N contacts          0.200 Angs
    Sigma for HBOND pairs           0.100 Angs (contacts between HB_types A and D or B's)
    Contact distance increment for each atom in HBOND          -0.150 Angs 
    Contact distance increment for H-bond acceptor              0.100 Angs 
    Contact distance increment for 1-4 with an O atom          -0.050 Angs 
    Contact distance increment for 1-4 with an N atom          -0.180 Angs 
    Contact distance increment for 1-4 with an C atom          -0.370 Angs 
    Contact distance increment for 1-4 with an S atom          -0.150 Angs 
    Contact distance increment for 1-4 with an P atom          -0.150 Angs 
    Contact distance increment for 1-4 with any other atom     -0.150 Angs 
    All atoms matched for CCP4 atom type.
    Number of CCP4 atom types used is         24 (H-bound and not H-bound counted separately)

 Setting up short/ideal-distance contacts
   Using gelly special position handling. (For TNT instead rerun with argument '-special_tnt')

   No command line argument '-special_all' specified. We will auto-generate
   EXCLUDE cards only for atom-atom contacts across symmetry (i.e. on special
   position) when this involves a contact within a residue to itself.

   Have set up non-bonded exclusions - 1-3 contacts
   Atom A|27:CA         makes the most 1-3 contacts:  6

   Have set up non-bonded exclusions - 1-4 contacts
   Atom A|27:N          makes the most 1-4 contacts:  7

   Have set up non-bonded exclusions - 1-5 contacts
   Atom A|28:C.A        makes the most 1-5 contacts: 10

 ############################################################################################################
 ###################################################### setting up contact terms
 ############################################################################################################


 ============= Call Gelly versions of TNT functions

 WARNING: 1-4 contact without 1-(2,3)-4 data for A|401:O2(CIT)        and A|401:C3(CIT)        X-X
 WARNING: 1-4 contact without 1-(2,3)-4 data for A|401:C2(CIT)        and A|401:O6(CIT)        X-X
 WARNING: 1-4 contact without 1-(2,3)-4 data for A|401:O7(CIT)        and A|401:O5(CIT)        X-X
 WARNING: 1-4 contact without 1-(2,3)-4 data for A|401:C4(CIT)        and A|401:O6(CIT)        X-X
 WARNING: 1-4 contact without 1-(2,3)-4 data for B|401:N02(LIG)       and B|401:N05(LIG)       X-X
 WARNING: 1-4 contact without 1-(2,3)-4 data for B|401:C03(LIG)       and B|401:C06(LIG)       X-X
 WARNING: 1-4 contact without 1-(2,3)-4 data for B|401:C04(LIG)       and B|401:C07(LIG)       X-X

       TERM      weight      number      TNT_fn    Gelly_fn        diff
       BOND       2.000        5660    1465.265    1465.265       0.000
       ANGL       2.000        7681    5271.375    5271.375       0.000
       IMPR       0.000           0       0.000       0.000       0.000
       TORS       2.000        1968    3083.611    3083.611      -0.000
       TRIG       2.000           0       0.000       0.000       0.000
       PLAN       5.000         964    2536.682    2536.682       0.000
       CONT       5.000           0     203.370       0.000     203.370
       BCOR      10.000        5630    5142.775    4854.116     288.659


 ============= Call Gelly geometry functions

   Switching torsion functional form to sinusoidal rather than split harmonic functional.
      if you want this not to happen then rerun with '-torsharm'
      Weight for sinusoidal torsion=     2.000
   BCOR function/gradient will be split between H and non-H atoms (as for any atom pair). Use '-bcorrel_all_in_hydrogens' to change this.

 ----------- Fix B-correlation restraints

 
 Checking for bonds that do not have a bcorrel restraint defined
 We will wipe all existing BCORrels if OCC=0.0 atom(s) is/are involved. This can be' switched off via the '-bcorrel_nowipe_zeroocc' flag.
 We will wipe all existing BCORrels if only non-hydrogen atom(s) with fixed B(iso) are involved. This can be switched off via the '-bcorrel_nowipe_fixb_nonhydrogens' flag.
 We will keep all existing BCORrels if hydrogen atom(s) with fixed B(iso) is/are involved. This can be switched off via the '-bcorrel_wipe_fixb_hydrogens' flag.
 No -bcorrel_missing_sigma found. So will set sigma to     2.5 Ang**2 for any bond with no existing BCORrel
 No -bcorrel_missing_sigma_hydrogens found. So will set sigma to     1.0 Ang**2 for any bond with no existing BCORrel involving hydrogens

 No active BCORels found involving OCC=0.0 atom(s)


 No active BCORels found involving fixed-B (only) non-hydrogen atom(s)

    add a default BCOR for bond A|401:C1(CIT) A|401:C2(CIT)
    add a default BCOR for bond A|401:C1(CIT) A|401:O1(CIT)
    add a default BCOR for bond A|401:C1(CIT) A|401:O2(CIT)
    add a default BCOR for bond A|401:C2(CIT) A|401:C3(CIT)
    add a default BCOR for bond A|401:C3(CIT) A|401:C4(CIT)
    add a default BCOR for bond A|401:C3(CIT) A|401:C6(CIT)
    add a default BCOR for bond A|401:C3(CIT) A|401:O7(CIT)
    add a default BCOR for bond A|401:C4(CIT) A|401:C5(CIT)
    add a default BCOR for bond A|401:C5(CIT) A|401:O3(CIT)
    add a default BCOR for bond A|401:C5(CIT) A|401:O4(CIT)
    add a default BCOR for bond A|401:C6(CIT) A|401:O5(CIT)
    add a default BCOR for bond A|401:C6(CIT) A|401:O6(CIT)
    add a default BCOR for bond B|401:C01(LIG) B|401:N02(LIG)
    add a default BCOR for bond B|401:N02(LIG) B|401:C03(LIG)
    add a default BCOR for bond B|401:N02(LIG) B|401:C07(LIG)
    add a default BCOR for bond B|401:C03(LIG) B|401:C04(LIG)
    add a default BCOR for bond B|401:C04(LIG) B|401:N05(LIG)
    add a default BCOR for bond B|401:N05(LIG) B|401:C06(LIG)
    add a default BCOR for bond B|401:N05(LIG) B|401:C08(LIG)
    add a default BCOR for bond B|401:C06(LIG) B|401:C07(LIG)
    add a default BCOR for bond B|401:C08(LIG) B|401:C09(LIG)
    add a default BCOR for bond B|401:C08(LIG) B|401:C17(LIG)
    add a default BCOR for bond B|401:C09(LIG) B|401:C10(LIG)
    add a default BCOR for bond B|401:C10(LIG) B|401:C11(LIG)
    add a default BCOR for bond B|401:C11(LIG) B|401:N12(LIG)
    add a default BCOR for bond B|401:C11(LIG) B|401:C16(LIG)
    add a default BCOR for bond B|401:N12(LIG) B|401:C13(LIG)
    add a default BCOR for bond B|401:C13(LIG) B|401:O14(LIG)
    add a default BCOR for bond B|401:C13(LIG) B|401:C15(LIG)
    add a default BCOR for bond B|401:C16(LIG) B|401:C17(LIG)

 Have added a total of        30 BCORrels for bonds which do not already have a BCORrel


 No bonds (for potential BCORrel addition) were skipped due to both atoms having constant/fixed B(iso)


 ----------- Deactivate hydrogen-free planes if hydrogen atoms present

 A total of       0 planes have been deactivated because they are duplicated hydrogen-free and with-hydrogen forms.

 ----------- Handle peptide omega angle(s)

      Torsion angles split into peptide omega and other
         Number of peptide omega angles is       698
         Number of other torsion is             1270
      Peptide omega angles are monitored rather than directly restrained (PLANE used instead).

 ----------- Fix cis-PRO

 Now apply the fix to get correct restraints for cis PRO residues
   Have got      11 bonds records and      26 angles
   from GEOMETRY cards for the special PROC residue
   Weight for ideal-distance contact term=     4.000

 ----------- Prepare SSBOND/LINK for output PDB file


 ----------- Prepare for QM


 ----------- Prepare symmetry in utility distance restraints


 ############### Setting up Rhofit contacts (if needed)

 No ligand-(fixed protein) contact term will be used

 ############### Working out initial function and gradient


   MaxLik Scaling Cycle 0002
 
 Initial functional value = 6.122080E+05 rms gradient=      17.9846
 X-ray contribution to function value = 5.761930E+05
 Geometry contribution (inc ncs)      =      36015.0
 Geometry function summary
     term                           number     weight       rms        GooF      function     fn/numb
     ================================================================================================
     BOND bond lengths (angs)       5660       2.000      0.0072       0.360      1465.27       0.259
     ANGL bond angles (degs)        7681       2.000        0.98       0.586      5271.37       0.686
     TORS split-harmonic tors       1270       0.000       17.51       1.102         0.00       0.000
     SINTOR sinusoidal tors         1270       2.000       17.51                  2391.99       1.883
     PLAN general plane (angs)       964       5.000      0.0145       0.725      2536.68       2.631
     BCORrelations (angs^2)         5660      10.000       0.821       0.293      4854.72       0.858
     CONT (bad contacts)               0       5.000                                 0.00
     IDEAL(ideal-dist contact)      5069       4.000                             10105.90
     CHIRAL (gelly semiharmon)       740       5.000                                45.61
     SIM similarity restraint for NCS and/or TARGET                               9343.46
     OCCSUM restraint on sum of occupanies                                           0.00       as none defined
     DISTAN utility restraints interatomic distance                                  0.00       as none defined
     UTILANGLE utility angle restraints                                              0.00       as none defined
     UTILTOR utility restraints on torsion angles                                    0.00       as none defined
     Peptide omega torsion angles                           3.01 degs - monitor only
 
 WARNING: Have |delta/sigma| deviations > 5.0 sigma. Number of outliers for each term: 
 WARNING:         4 bond angles.  Worst is     5.4 sigs   135.31 degs A|28:C.B(ASN)=A|29:N(ASN)=A|29:CA(ASN)

 Weighted rms      0.0072         0.983  (temporary rms bond and angle for BUSTER to pick up!)
 Weighted intra-residual rms(bond) for StdProtein (excluding H)      0.0070
 Weighted intra-residual rms(bond) for Buffer     (excluding H)      0.0034

 ############### Running initial check


 Initial Geometry Sanity Check

 Check for duplicate bonds: no duplicates found
 
 Check for duplicate or missing bond angles: all are OK
 Check for bond between an atom and itself not involving symmetry:  all are OK
   There are no bad contacts where Delta/sigma is greater than     6.5
   Worst contact dist=   3.002 std=   3.802 sigma=   0.206 abs(Delta/sigma)=    3.885 ats A|104:CE (MET) B|23:CB (PRO)
   It is likely that the geometry libraries, sequence info and links match the input molecule
 GEOMETRY CHECK OVERALL:  PASS


 ############### Creating geometry/screen report


 ############### Option "tao" - run Tao optimizer

 TAO: Toolkit for Advanced Optimization. Reference: 
     Munson, T., Sarich, J., Wild, S., Benson, S., McInnes, L.C. (2018)
     "TAO User Manual (Revision 3.9)", Mathematics and Computer Science
     Division, Argonne National Laboratory
     ANL/MCS-TM-322, http://www.mcs.anl.gov/petsc/
 
 Have found -keeppdb flag so will write out a .pdb file every 1000000 its during opt
 Will apply TAO optimization 
 Maximum number of iterations      100
 Convergence test on Grms       4.0000 (-glim)
 Limit on rms displacement to original position (rmsD2init): NONE test inactive (-dlim)

    analysing argument no 0 : -autoncs with j=0
    analysing argument no 1 : -hydrogens_input_occ2zero with j=0
    analysing argument no 2 : -hydrogens_output_occ2heavy with j=0
    analysing argument no 3 : -screen with j=0
    analysing argument no 4 : 100 with j=0
    analysing argument no 5 : -screen_sigma with j=0
    analysing argument no 6 : 3.0 with j=0
    analysing argument no 7 : -keeppdb with j=0
    analysing argument no 8 : 1000000 with j=0
    analysing argument no 9 : -taomonitor_verbose with j=0
    found argument starting with "-tao" : -taomonitor_verbose
    analysing argument no 10 : 2 with j=1
    found value after -tao/-options_/-viewer_ argument : "2"
 PETSC/TAO: setting option "-taomonitor_verbose" = "2"


   MaxLik Scaling Cycle 0003
   Iters     Tot_Funct    Xray_Funct    Geom_Funct          Grms     rmsD2init     maxD2init             R         Rfree           LLG       LLGfree     rms(bond)    rms(angle)       Opttag_H
   Iters             K   B_IMPF_FRAG        K_MISS   B_TEMP_MISS   K_IMPF_MISS   B_IMPF_MISS        K_SOLV   B_TEMP_SOLV   K_IMPF_SOLV   B_IMPF_SOLV        B_TEMP     B_TEMP_11     B_TEMP_12     B_TEMP_22     B_TEMP_13     B_TEMP_23     B_TEMP_33      K_WILSON      B_WILSON      FGCYCLES       Scatag_H
   Iters          BOND         ANGLE      IMPROPER       TORSION    SINTORSION      TRIGONAL        PLANAR  BCORRELATION       CONTACT     IDEALDIST        CHIRAL    SIMILARITY       OCCSSUM  UTILDISTANCE     UTILANGLE   UTILTORSION        RHOFIT        ADP-01        ADP-02        ADP-03        ADP-04        ADP-05        ADP-06        ADP-07        ADP-08        ADP-09        ADP-10      RHOFITPC            QM       Funtag_H
       0  6.122080E+05  5.761930E+05       36015.0       17.9846  0.000000E+00  0.000000E+00      0.199961      0.227513  0.000000E+00  0.000000E+00    0.00721936      0.983295       Opttag_D
       0       59.5092       2.27880       1.00000  0.000000E+00       1.00000  0.000000E+00      0.886998       55.4536       1.00000       20.0065  0.000000E+00       1.78846  0.000000E+00       1.78846  0.000000E+00  0.000000E+00      -3.57692       59.6917       1.81913        2       Scatag_D
       0       1465.27       5271.37  0.000000E+00  0.000000E+00       2391.99  0.000000E+00       2536.68       4854.72  0.000000E+00       10105.9       45.6130       9343.46 -1.000000E+10 -1.000000E+10 -1.000000E+10 -1.000000E+10 -1.000000E+10  0.000000E+00  0.000000E+00  0.000000E+00  0.000000E+00  0.000000E+00  0.000000E+00  0.000000E+00  0.000000E+00  0.000000E+00  0.000000E+00  0.000000E+00  0.000000E+00       Funtag_D
   Geometry Restraint Deviations SCREEN Information

 

 WARNING: have displacement of 27.37 Angs from initial (> 15.00 Angs)
          for variable number= 850 (type Y        ; returned function value as   5.895893E+010 )
          Involving atom A|27:CG2

 An atom has moved more than     1.50 A from the initial/last
 position - if this was an accepted trial point, probably need to update the
 non-bonded contact list.


   MaxLik Scaling Cycle 0004

   MaxLik Scaling Cycle 0005

   MaxLik Scaling Cycle 0006

   Wilson plot

   Statistics in resolution bins after refinement cycle 0001
```


|  |  |
| --- | --- |
|  | - Rxpct factors plot- Rxpct factors table- <Log-Lik Gain>- Ln Sigma A- Correlation Coefficients |


```
   Iters     Tot_Funct    Xray_Funct    Geom_Funct          Grms     rmsD2init     maxD2init             R         Rfree           LLG       LLGfree     rms(bond)    rms(angle)
   Iters             K   B_IMPF_FRAG        K_MISS   B_TEMP_MISS   K_IMPF_MISS   B_IMPF_MISS        K_SOLV   B_TEMP_SOLV   K_IMPF_SOLV   B_IMPF_SOLV        B_TEMP     B_TEMP_11     B_TEMP_12     B_TEMP_22     B_TEMP_13     B_TEMP_23     B_TEMP_33      K_WILSON      B_WILSON      FGCYCLES
   Iters          BOND         ANGLE      IMPROPER       TORSION    SINTORSION      TRIGONAL        PLANAR  BCORRELATION       CONTACT     IDEALDIST        CHIRAL    SIMILARITY       OCCSSUM  UTILDISTANCE     UTILANGLE   UTILTORSION        RHOFIT        ADP-01        ADP-02        ADP-03        ADP-04        ADP-05        ADP-06        ADP-07        ADP-08        ADP-09        ADP-10      RHOFITPC            QM
       1  6.121095E+05  5.758414E+05       36268.0       24.1092  6.823068E-04    0.00486461      0.199783      0.227483  7.760535E-04  2.007027E-04    0.00731669      0.985608       Opttag_D
       1       59.4933       2.26433       1.00000  0.000000E+00       1.00000  0.000000E+00      0.887017       55.3341       1.00000       20.0141  0.000000E+00       1.79027  0.000000E+00       1.79027  0.000000E+00  0.000000E+00      -3.58054       59.6917       1.81913        3       Scatag_D
       1       1505.04       5296.20  0.000000E+00  0.000000E+00       2392.19  0.000000E+00       2543.84       5043.56  0.000000E+00       10103.8       46.7828       9336.62 -1.000000E+10 -1.000000E+10 -1.000000E+10 -1.000000E+10 -1.000000E+10  0.000000E+00  0.000000E+00  0.000000E+00  0.000000E+00  0.000000E+00  0.000000E+00  0.000000E+00  0.000000E+00  0.000000E+00  0.000000E+00  0.000000E+00  0.000000E+00       Funtag_D
   Geometry Restraint Deviations SCREEN Information

 

   MaxLik Scaling Cycle 0007

   Wilson plot

   Statistics in resolution bins after refinement cycle 0002
```


|  |  |
| --- | --- |
|  | - Rxpct factors plot- Rxpct factors table- <Log-Lik Gain>- Ln Sigma A- Correlation Coefficients |


```
   Iters     Tot_Funct    Xray_Funct    Geom_Funct          Grms     rmsD2init     maxD2init             R         Rfree           LLG       LLGfree     rms(bond)    rms(angle)
   Iters             K   B_IMPF_FRAG        K_MISS   B_TEMP_MISS   K_IMPF_MISS   B_IMPF_MISS        K_SOLV   B_TEMP_SOLV   K_IMPF_SOLV   B_IMPF_SOLV        B_TEMP     B_TEMP_11     B_TEMP_12     B_TEMP_22     B_TEMP_13     B_TEMP_23     B_TEMP_33      K_WILSON      B_WILSON      FGCYCLES
   Iters          BOND         ANGLE      IMPROPER       TORSION    SINTORSION      TRIGONAL        PLANAR  BCORRELATION       CONTACT     IDEALDIST        CHIRAL    SIMILARITY       OCCSSUM  UTILDISTANCE     UTILANGLE   UTILTORSION        RHOFIT        ADP-01        ADP-02        ADP-03        ADP-04        ADP-05        ADP-06        ADP-07        ADP-08        ADP-09        ADP-10      RHOFITPC            QM
       2  6.119666E+05  5.754572E+05       36509.4       26.2587    0.00184482     0.0173946      0.199547      0.227444    0.00162389  3.761106E-04    0.00745533      0.988782       Opttag_D
       2       59.4745       2.25002       1.00000  0.000000E+00       1.00000  0.000000E+00      0.887063       55.2350       1.00000       19.9745  0.000000E+00       1.78303  0.000000E+00       1.78303  0.000000E+00  0.000000E+00      -3.56606       59.6917       1.81913        1       Scatag_D
       2       1562.62       5330.37  0.000000E+00  0.000000E+00       2392.78  0.000000E+00       2559.01       5172.12  0.000000E+00       10107.1       47.4501       9337.90 -1.000000E+10 -1.000000E+10 -1.000000E+10 -1.000000E+10 -1.000000E+10  0.000000E+00  0.000000E+00  0.000000E+00  0.000000E+00  0.000000E+00  0.000000E+00  0.000000E+00  0.000000E+00  0.000000E+00  0.000000E+00  0.000000E+00  0.000000E+00       Funtag_D
   Geometry Restraint Deviations SCREEN Information

 
       3  6.118339E+05  5.750517E+05       36782.2       26.5470    0.00338354     0.0352126      0.199284      0.227390    0.00251874  6.045172E-04    0.00760755      0.993226       Opttag_D
       3       59.4525       2.23541       1.00000  0.000000E+00       1.00000  0.000000E+00      0.887129       55.1343       1.00000       19.9198  0.000000E+00       1.77315  0.000000E+00       1.77315  0.000000E+00  0.000000E+00      -3.54630       59.6917       1.81913        1       Scatag_D
       3       1627.08       5378.38  0.000000E+00  0.000000E+00       2393.90  0.000000E+00       2580.13       5284.95  0.000000E+00       10117.7       47.5334       9352.55 -1.000000E+10 -1.000000E+10 -1.000000E+10 -1.000000E+10 -1.000000E+10  0.000000E+00  0.000000E+00  0.000000E+00  0.000000E+00  0.000000E+00  0.000000E+00  0.000000E+00  0.000000E+00  0.000000E+00  0.000000E+00  0.000000E+00  0.000000E+00       Funtag_D
       4  6.117226E+05  5.746593E+05       37063.3       28.1223    0.00510958     0.0568239      0.199029      0.227339    0.00338462  8.121459E-04    0.00774554      0.998184       Opttag_D
       4       59.4293       2.22145       1.00000  0.000000E+00       1.00000  0.000000E+00      0.887214       55.0334       1.00000       19.8590  0.000000E+00       1.76264  0.000000E+00       1.76264  0.000000E+00  0.000000E+00      -3.52528       59.6917       1.81913        1       Scatag_D
       4       1686.64       5432.21  0.000000E+00  0.000000E+00       2395.38  0.000000E+00       2604.11       5387.30  0.000000E+00       10136.3       47.3124       9374.05 -1.000000E+10 -1.000000E+10 -1.000000E+10 -1.000000E+10 -1.000000E+10  0.000000E+00  0.000000E+00  0.000000E+00  0.000000E+00  0.000000E+00  0.000000E+00  0.000000E+00  0.000000E+00  0.000000E+00  0.000000E+00  0.000000E+00  0.000000E+00       Funtag_D
       5  6.116161E+05  5.743886E+05       37227.5       23.9670    0.00657525     0.0767852      0.198849      0.227301    0.00398191  9.378674E-04    0.00780214       1.00088       Opttag_D
       5       59.4110       2.21199       1.00000  0.000000E+00       1.00000  0.000000E+00      0.887291       54.9636       1.00000       19.8085  0.000000E+00       1.75442  0.000000E+00       1.75442  0.000000E+00  0.000000E+00      -3.50884       59.6917       1.81913        1       Scatag_D
       5       1711.38       5461.63  0.000000E+00  0.000000E+00       2396.94  0.000000E+00       2622.84       5444.81  0.000000E+00       10156.8       47.0327       9386.05 -1.000000E+10 -1.000000E+10 -1.000000E+10 -1.000000E+10 -1.000000E+10  0.000000E+00  0.000000E+00  0.000000E+00  0.000000E+00  0.000000E+00  0.000000E+00  0.000000E+00  0.000000E+00  0.000000E+00  0.000000E+00  0.000000E+00  0.000000E+00       Funtag_D
       6  6.114953E+05  5.741523E+05       37343.0       22.1866    0.00828627      0.101576      0.198692      0.227277    0.00450335    0.00100397    0.00780639       1.00381       Opttag_D
       6       59.3924       2.20391       1.00000  0.000000E+00       1.00000  0.000000E+00      0.887379       54.9065       1.00000       19.7532  0.000000E+00       1.74679  0.000000E+00       1.74679  0.000000E+00  0.000000E+00      -3.49357       59.6917       1.81913        1       Scatag_D
       6       1713.24       5493.58  0.000000E+00  0.000000E+00       2399.01  0.000000E+00       2640.28       5478.73  0.000000E+00       10185.1       46.9550       9386.06 -1.000000E+10 -1.000000E+10 -1.000000E+10 -1.000000E+10 -1.000000E+10  0.000000E+00  0.000000E+00  0.000000E+00  0.000000E+00  0.000000E+00  0.000000E+00  0.000000E+00  0.000000E+00  0.000000E+00  0.000000E+00  0.000000E+00  0.000000E+00       Funtag_D
       7  6.113687E+05  5.738802E+05       37488.6       28.4908     0.0108742      0.139146      0.198508      0.227290    0.00510381  9.041409E-04    0.00778337       1.00857       Opttag_D
       7       59.3701       2.19478       1.00000  0.000000E+00       1.00000  0.000000E+00      0.887500       54.8512       1.00000       19.6775  0.000000E+00       1.73940  0.000000E+00       1.73940  0.000000E+00  0.000000E+00      -3.47880       59.6917       1.81913        1       Scatag_D
       7       1703.16       5545.84  0.000000E+00  0.000000E+00       2402.09  0.000000E+00       2660.04       5519.64  0.000000E+00       10231.3       47.3974       9379.04 -1.000000E+10 -1.000000E+10 -1.000000E+10 -1.000000E+10 -1.000000E+10  0.000000E+00  0.000000E+00  0.000000E+00  0.000000E+00  0.000000E+00  0.000000E+00  0.000000E+00  0.000000E+00  0.000000E+00  0.000000E+00  0.000000E+00  0.000000E+00       Funtag_D
       8  6.112683E+05  5.736838E+05       37584.5       28.8213     0.0130585      0.169952      0.198373      0.227331    0.00553719  6.785950E-04    0.00776619       1.01164       Opttag_D
       8       59.3565       2.18833       1.00000  0.000000E+00       1.00000  0.000000E+00      0.887595       54.8191       1.00000       19.6187  0.000000E+00       1.73518  0.000000E+00       1.73518  0.000000E+00  0.000000E+00      -3.47035       59.6917       1.81913        1       Scatag_D
       8       1695.64       5579.67  0.000000E+00  0.000000E+00       2404.90  0.000000E+00       2673.30       5536.93  0.000000E+00       10270.8       48.0240       9375.22 -1.000000E+10 -1.000000E+10 -1.000000E+10 -1.000000E+10 -1.000000E+10  0.000000E+00  0.000000E+00  0.000000E+00  0.000000E+00  0.000000E+00  0.000000E+00  0.000000E+00  0.000000E+00  0.000000E+00  0.000000E+00  0.000000E+00  0.000000E+00       Funtag_D
       9  6.111765E+05  5.736300E+05       37546.5       19.2145     0.0138606      0.181082      0.198338      0.227371    0.00565586  4.997242E-04    0.00773955       1.01023       Opttag_D
       9       59.3558       2.18678       1.00000  0.000000E+00       1.00000  0.000000E+00      0.887623       54.8230       1.00000       19.5988  0.000000E+00       1.73596  0.000000E+00       1.73596  0.000000E+00  0.000000E+00      -3.47192       59.6917       1.81913        1       Scatag_D
       9       1684.03       5564.08  0.000000E+00  0.000000E+00       2405.96  0.000000E+00       2673.28       5522.18  0.000000E+00       10282.7       48.3980       9365.88 -1.000000E+10 -1.000000E+10 -1.000000E+10 -1.000000E+10 -1.000000E+10  0.000000E+00  0.000000E+00  0.000000E+00  0.000000E+00  0.000000E+00  0.000000E+00  0.000000E+00  0.000000E+00  0.000000E+00  0.000000E+00  0.000000E+00  0.000000E+00       Funtag_D
      10  6.110763E+05  5.735081E+05       37568.3       18.6670     0.0153622      0.200722      0.198255      0.227435    0.00592497  1.910666E-04    0.00773412       1.01080       Opttag_D
      10       59.3570       2.18334       1.00000  0.000000E+00       1.00000  0.000000E+00      0.887677       54.8258       1.00000       19.5619  0.000000E+00       1.73749  0.000000E+00       1.73749  0.000000E+00  0.000000E+00      -3.47498       59.6917       1.81913        1       Scatag_D
      10       1681.67       5570.44  0.000000E+00  0.000000E+00       2407.92  0.000000E+00       2674.80       5525.86  0.000000E+00       10302.9       48.9094       9355.71 -1.000000E+10 -1.000000E+10 -1.000000E+10 -1.000000E+10 -1.000000E+10  0.000000E+00  0.000000E+00  0.000000E+00  0.000000E+00  0.000000E+00  0.000000E+00  0.000000E+00  0.000000E+00  0.000000E+00  0.000000E+00  0.000000E+00  0.000000E+00       Funtag_D
      11  6.110005E+05  5.733008E+05       37699.6       28.4189     0.0179815      0.233656      0.198112      0.227504    0.00638224 -2.567128E-04    0.00775323       1.01357       Opttag_D
      11       59.3606       2.17779       1.00000  0.000000E+00       1.00000  0.000000E+00      0.887782       54.8225       1.00000       19.4998  0.000000E+00       1.73923  0.000000E+00       1.73923  0.000000E+00  0.000000E+00      -3.47846       59.6917       1.81913        1       Scatag_D
      11       1689.99       5600.99  0.000000E+00  0.000000E+00       2411.42  0.000000E+00       2682.20       5575.29  0.000000E+00       10338.0       49.5371       9352.22 -1.000000E+10 -1.000000E+10 -1.000000E+10 -1.000000E+10 -1.000000E+10  0.000000E+00  0.000000E+00  0.000000E+00  0.000000E+00  0.000000E+00  0.000000E+00  0.000000E+00  0.000000E+00  0.000000E+00  0.000000E+00  0.000000E+00  0.000000E+00       Funtag_D
      12  6.109069E+05  5.731686E+05       37738.3       19.0966     0.0196868      0.254331      0.198019      0.227531    0.00667396 -5.033377E-04    0.00776229       1.01430       Opttag_D
      12       59.3651       2.17447       1.00000  0.000000E+00       1.00000  0.000000E+00      0.887853       54.8187       1.00000       19.4607  0.000000E+00       1.74046  0.000000E+00       1.74046  0.000000E+00  0.000000E+00      -3.48091       59.6917       1.81913        1       Scatag_D
      12       1693.94       5609.09  0.000000E+00  0.000000E+00       2413.74  0.000000E+00       2688.26       5571.51  0.000000E+00       10359.3       49.7992       9352.72 -1.000000E+10 -1.000000E+10 -1.000000E+10 -1.000000E+10 -1.000000E+10  0.000000E+00  0.000000E+00  0.000000E+00  0.000000E+00  0.000000E+00  0.000000E+00  0.000000E+00  0.000000E+00  0.000000E+00  0.000000E+00  0.000000E+00  0.000000E+00       Funtag_D
      13  6.108235E+05  5.730245E+05       37799.0       17.1466     0.0215749      0.276221      0.197916      0.227541    0.00699210 -7.443918E-04    0.00776806       1.01566       Opttag_D
      13       59.3729       2.17118       1.00000  0.000000E+00       1.00000  0.000000E+00      0.887938       54.8140       1.00000       19.4174  0.000000E+00       1.74202  0.000000E+00       1.74202  0.000000E+00  0.000000E+00      -3.48404       59.6917       1.81913        1       Scatag_D
      13       1696.46       5624.10  0.000000E+00  0.000000E+00       2416.35  0.000000E+00       2695.71       5584.08  0.000000E+00       10379.3       49.9719       9353.00 -1.000000E+10 -1.000000E+10 -1.000000E+10 -1.000000E+10 -1.000000E+10  0.000000E+00  0.000000E+00  0.000000E+00  0.000000E+00  0.000000E+00  0.000000E+00  0.000000E+00  0.000000E+00  0.000000E+00  0.000000E+00  0.000000E+00  0.000000E+00       Funtag_D
      14  6.107523E+05  5.728412E+05       37911.1       23.1839     0.0242363      0.305950      0.197783      0.227563    0.00739652   -0.00109686    0.00778778       1.01849       Opttag_D
      14       59.3858       2.16726       1.00000  0.000000E+00       1.00000  0.000000E+00      0.888061       54.8089       1.00000       19.3589  0.000000E+00       1.74473  0.000000E+00       1.74473  0.000000E+00  0.000000E+00      -3.48945       59.6917       1.81913        1       Scatag_D
      14       1705.09       5655.43  0.000000E+00  0.000000E+00       2420.01  0.000000E+00       2706.82       5613.15  0.000000E+00       10405.5       50.3062       9354.82 -1.000000E+10 -1.000000E+10 -1.000000E+10 -1.000000E+10 -1.000000E+10  0.000000E+00  0.000000E+00  0.000000E+00  0.000000E+00  0.000000E+00  0.000000E+00  0.000000E+00  0.000000E+00  0.000000E+00  0.000000E+00  0.000000E+00  0.000000E+00       Funtag_D
      15  6.106770E+05  5.727053E+05       37971.7       17.8920     0.0264287      0.329420      0.197685      0.227591    0.00769650   -0.00138535    0.00780521       1.01984       Opttag_D
      15       59.3974       2.16451       1.00000  0.000000E+00       1.00000  0.000000E+00      0.888160       54.8052       1.00000       19.3135  0.000000E+00       1.74737  0.000000E+00       1.74737  0.000000E+00  0.000000E+00      -3.49473       59.6917       1.81913        1       Scatag_D
      15       1712.73       5670.47  0.000000E+00  0.000000E+00       2423.03  0.000000E+00       2716.18       5615.72  0.000000E+00       10426.1       50.6168       9356.87 -1.000000E+10 -1.000000E+10 -1.000000E+10 -1.000000E+10 -1.000000E+10  0.000000E+00  0.000000E+00  0.000000E+00  0.000000E+00  0.000000E+00  0.000000E+00  0.000000E+00  0.000000E+00  0.000000E+00  0.000000E+00  0.000000E+00  0.000000E+00       Funtag_D
      16  6.106117E+05  5.726138E+05       37997.9       15.7489     0.0280046      0.344844      0.197621      0.227627    0.00789826   -0.00158695    0.00781040       1.02062       Opttag_D
      16       59.4060       2.16265       1.00000  0.000000E+00       1.00000  0.000000E+00      0.888229       54.8007       1.00000       19.2819  0.000000E+00       1.74949  0.000000E+00       1.74949  0.000000E+00  0.000000E+00      -3.49899       59.6917       1.81913        1       Scatag_D
      16       1715.00       5679.14  0.000000E+00  0.000000E+00       2425.15  0.000000E+00       2722.15       5612.82  0.000000E+00       10438.0       50.8315       9354.81 -1.000000E+10 -1.000000E+10 -1.000000E+10 -1.000000E+10 -1.000000E+10  0.000000E+00  0.000000E+00  0.000000E+00  0.000000E+00  0.000000E+00  0.000000E+00  0.000000E+00  0.000000E+00  0.000000E+00  0.000000E+00  0.000000E+00  0.000000E+00       Funtag_D
      17  6.105482E+05  5.724990E+05       38049.3       18.6798     0.0301748      0.364986      0.197540      0.227689    0.00815174   -0.00185767    0.00782615       1.02139       Opttag_D
      17       59.4158       2.16010       1.00000  0.000000E+00       1.00000  0.000000E+00      0.888319       54.7897       1.00000       19.2407  0.000000E+00       1.75203  0.000000E+00       1.75203  0.000000E+00  0.000000E+00      -3.50406       59.6917       1.81913        1       Scatag_D
      17       1721.93       5687.70  0.000000E+00  0.000000E+00       2428.16  0.000000E+00       2729.77       5624.32  0.000000E+00       10454.5       51.1847       9351.66 -1.000000E+10 -1.000000E+10 -1.000000E+10 -1.000000E+10 -1.000000E+10  0.000000E+00  0.000000E+00  0.000000E+00  0.000000E+00  0.000000E+00  0.000000E+00  0.000000E+00  0.000000E+00  0.000000E+00  0.000000E+00  0.000000E+00  0.000000E+00       Funtag_D
      18  6.104926E+05  5.724267E+05       38065.9       15.9269     0.0316817      0.378338      0.197495      0.227737    0.00831106   -0.00205190    0.00783453       1.02157       Opttag_D
      18       59.4201       2.15826       1.00000  0.000000E+00       1.00000  0.000000E+00      0.888373       54.7789       1.00000       19.2134  0.000000E+00       1.75331  0.000000E+00       1.75331  0.000000E+00  0.000000E+00      -3.50662       59.6917       1.81913        1       Scatag_D
      18       1725.62       5689.78  0.000000E+00  0.000000E+00       2430.37  0.000000E+00       2734.09       5618.63  0.000000E+00       10466.9       51.4719       9349.03 -1.000000E+10 -1.000000E+10 -1.000000E+10 -1.000000E+10 -1.000000E+10  0.000000E+00  0.000000E+00  0.000000E+00  0.000000E+00  0.000000E+00  0.000000E+00  0.000000E+00  0.000000E+00  0.000000E+00  0.000000E+00  0.000000E+00  0.000000E+00       Funtag_D
      19  6.104387E+05  5.723811E+05       38057.6       13.7051     0.0327557      0.387230      0.197469      0.227777    0.00841169   -0.00218545    0.00782751       1.02117       Opttag_D
      19       59.4200       2.15683       1.00000  0.000000E+00       1.00000  0.000000E+00      0.888403       54.7666       1.00000       19.1939  0.000000E+00       1.75357  0.000000E+00       1.75357  0.000000E+00  0.000000E+00      -3.50714       59.6917       1.81913        1       Scatag_D
      19       1722.53       5685.28  0.000000E+00  0.000000E+00       2432.09  0.000000E+00       2735.28       5610.81  0.000000E+00       10475.8       51.6940       9344.08 -1.000000E+10 -1.000000E+10 -1.000000E+10 -1.000000E+10 -1.000000E+10  0.000000E+00  0.000000E+00  0.000000E+00  0.000000E+00  0.000000E+00  0.000000E+00  0.000000E+00  0.000000E+00  0.000000E+00  0.000000E+00  0.000000E+00  0.000000E+00       Funtag_D
      20  6.103754E+05  5.723035E+05       38071.9       17.7056     0.0350359      0.406776      0.197421      0.227867    0.00858309   -0.00248944    0.00781764       1.02123       Opttag_D
      20       59.4191       2.15445       1.00000  0.000000E+00       1.00000  0.000000E+00      0.888463       54.7438       1.00000       19.1529  0.000000E+00       1.75413  0.000000E+00       1.75413  0.000000E+00  0.000000E+00      -3.50826       59.6917       1.81913        1       Scatag_D
      20       1718.19       5685.94  0.000000E+00  0.000000E+00       2435.84  0.000000E+00       2737.97       5607.25  0.000000E+00       10495.8       52.1524       9338.77 -1.000000E+10 -1.000000E+10 -1.000000E+10 -1.000000E+10 -1.000000E+10  0.000000E+00  0.000000E+00  0.000000E+00  0.000000E+00  0.000000E+00  0.000000E+00  0.000000E+00  0.000000E+00  0.000000E+00  0.000000E+00  0.000000E+00  0.000000E+00       Funtag_D
      21  6.103279E+05  5.722454E+05       38082.6       17.7371     0.0371561      0.425228      0.197380      0.227943    0.00871129   -0.00276181    0.00780757       1.02178       Opttag_D
      21       59.4188       2.15286       1.00000  0.000000E+00       1.00000  0.000000E+00      0.888516       54.7265       1.00000       19.1166  0.000000E+00       1.75480  0.000000E+00       1.75480  0.000000E+00  0.000000E+00      -3.50959       59.6917       1.81913        1       Scatag_D
      21       1713.76       5692.08  0.000000E+00  0.000000E+00       2439.44  0.000000E+00       2740.78       5591.95  0.000000E+00       10514.3       52.3965       9337.89 -1.000000E+10 -1.000000E+10 -1.000000E+10 -1.000000E+10 -1.000000E+10  0.000000E+00  0.000000E+00  0.000000E+00  0.000000E+00  0.000000E+00  0.000000E+00  0.000000E+00  0.000000E+00  0.000000E+00  0.000000E+00  0.000000E+00  0.000000E+00       Funtag_D
      22  6.102882E+05  5.722350E+05       38053.1       13.1302     0.0376403      0.429943      0.197370      0.227955    0.00873416   -0.00280878    0.00779101       1.02168       Opttag_D
      22       59.4181       2.15261       1.00000  0.000000E+00       1.00000  0.000000E+00      0.888525       54.7230       1.00000       19.1079  0.000000E+00       1.75498  0.000000E+00       1.75498  0.000000E+00  0.000000E+00      -3.50996       59.6917       1.81913        1       Scatag_D
      22       1706.50       5690.91  0.000000E+00  0.000000E+00       2440.24  0.000000E+00       2739.86       5572.39  0.000000E+00       10516.7       52.2446       9334.31 -1.000000E+10 -1.000000E+10 -1.000000E+10 -1.000000E+10 -1.000000E+10  0.000000E+00  0.000000E+00  0.000000E+00  0.000000E+00  0.000000E+00  0.000000E+00  0.000000E+00  0.000000E+00  0.000000E+00  0.000000E+00  0.000000E+00  0.000000E+00       Funtag_D
      23  6.102302E+05  5.721934E+05       38036.8       12.4472     0.0393702      0.446504      0.197339      0.228005    0.00882589   -0.00297907    0.00777527       1.02239       Opttag_D
      23       59.4186       2.15171       1.00000  0.000000E+00       1.00000  0.000000E+00      0.888563       54.7118       1.00000       19.0802  0.000000E+00       1.75577  0.000000E+00       1.75577  0.000000E+00  0.000000E+00      -3.51154       59.6917       1.81913        1       Scatag_D
      23       1699.61       5698.90  0.000000E+00  0.000000E+00       2442.98  0.000000E+00       2739.66       5547.92  0.000000E+00       10526.4       51.9784       9329.33 -1.000000E+10 -1.000000E+10 -1.000000E+10 -1.000000E+10 -1.000000E+10  0.000000E+00  0.000000E+00  0.000000E+00  0.000000E+00  0.000000E+00  0.000000E+00  0.000000E+00  0.000000E+00  0.000000E+00  0.000000E+00  0.000000E+00  0.000000E+00       Funtag_D
      24  6.101943E+05  5.721337E+05       38060.6       16.8999     0.0416062      0.467426      0.197298      0.228073    0.00895765   -0.00319287    0.00777627       1.02334       Opttag_D
      24       59.4209       2.15027       1.00000  0.000000E+00       1.00000  0.000000E+00      0.888610       54.6964       1.00000       19.0502  0.000000E+00       1.75670  0.000000E+00       1.75670  0.000000E+00  0.000000E+00      -3.51340       59.6917       1.81913        1       Scatag_D
      24       1700.05       5709.43  0.000000E+00  0.000000E+00       2446.44  0.000000E+00       2741.62       5543.64  0.000000E+00       10539.8       52.0836       9327.49 -1.000000E+10 -1.000000E+10 -1.000000E+10 -1.000000E+10 -1.000000E+10  0.000000E+00  0.000000E+00  0.000000E+00  0.000000E+00  0.000000E+00  0.000000E+00  0.000000E+00  0.000000E+00  0.000000E+00  0.000000E+00  0.000000E+00  0.000000E+00       Funtag_D
      25  6.101516E+05  5.720891E+05       38062.5       12.3701     0.0431916      0.482469      0.197270      0.228123    0.00905599   -0.00333501    0.00778182       1.02395       Opttag_D
      25       59.4237       2.14922       1.00000  0.000000E+00       1.00000  0.000000E+00      0.888643       54.6857       1.00000       19.0321  0.000000E+00       1.75704  0.000000E+00       1.75704  0.000000E+00  0.000000E+00      -3.51408       59.6917       1.81913        1       Scatag_D
      25       1702.48       5716.25  0.000000E+00  0.000000E+00       2448.80  0.000000E+00       2743.41       5525.17  0.000000E+00       10548.2       52.3203       9325.87 -1.000000E+10 -1.000000E+10 -1.000000E+10 -1.000000E+10 -1.000000E+10  0.000000E+00  0.000000E+00  0.000000E+00  0.000000E+00  0.000000E+00  0.000000E+00  0.000000E+00  0.000000E+00  0.000000E+00  0.000000E+00  0.000000E+00  0.000000E+00       Funtag_D
      26  6.101026E+05  5.720167E+05       38085.9       12.0232     0.0455859      0.510647      0.197228      0.228210    0.00921595   -0.00355528    0.00779944       1.02486       Opttag_D
      26       59.4297       2.14752       1.00000  0.000000E+00       1.00000  0.000000E+00      0.888685       54.6716       1.00000       19.0096  0.000000E+00       1.75716  0.000000E+00       1.75716  0.000000E+00  0.000000E+00      -3.51432       59.6917       1.81913        1       Scatag_D
      26       1710.20       5726.47  0.000000E+00  0.000000E+00       2452.30  0.000000E+00       2746.81       5516.52  0.000000E+00       10558.7       53.0701       9321.92 -1.000000E+10 -1.000000E+10 -1.000000E+10 -1.000000E+10 -1.000000E+10  0.000000E+00  0.000000E+00  0.000000E+00  0.000000E+00  0.000000E+00  0.000000E+00  0.000000E+00  0.000000E+00  0.000000E+00  0.000000E+00  0.000000E+00  0.000000E+00       Funtag_D
      27  6.100722E+05  5.719621E+05       38110.1       14.1391     0.0473869      0.538264      0.197198      0.228288    0.00933640   -0.00374439    0.00781061       1.02558       Opttag_D
      27       59.4351       2.14628       1.00000  0.000000E+00       1.00000  0.000000E+00      0.888709       54.6644       1.00000       18.9954  0.000000E+00       1.75697  0.000000E+00       1.75697  0.000000E+00  0.000000E+00      -3.51395       59.6917       1.81913        1       Scatag_D
      27       1715.10       5734.53  0.000000E+00  0.000000E+00       2454.94  0.000000E+00       2749.85       5518.52  0.000000E+00       10565.2       53.7707       9318.24 -1.000000E+10 -1.000000E+10 -1.000000E+10 -1.000000E+10 -1.000000E+10  0.000000E+00  0.000000E+00  0.000000E+00  0.000000E+00  0.000000E+00  0.000000E+00  0.000000E+00  0.000000E+00  0.000000E+00  0.000000E+00  0.000000E+00  0.000000E+00       Funtag_D
      28  6.100388E+05  5.719254E+05       38113.4       11.6002     0.0485613      0.556955      0.197179      0.228338    0.00941746   -0.00387779    0.00781298       1.02604       Opttag_D
      28       59.4391       2.14551       1.00000  0.000000E+00       1.00000  0.000000E+00      0.888719       54.6629       1.00000       18.9871  0.000000E+00       1.75659  0.000000E+00       1.75659  0.000000E+00  0.000000E+00      -3.51319       59.6917       1.81913        1       Scatag_D
      28       1716.14       5739.67  0.000000E+00  0.000000E+00       2456.74  0.000000E+00       2751.90       5511.63  0.000000E+00       10568.2       54.1229       9315.04 -1.000000E+10 -1.000000E+10 -1.000000E+10 -1.000000E+10 -1.000000E+10  0.000000E+00  0.000000E+00  0.000000E+00  0.000000E+00  0.000000E+00  0.000000E+00  0.000000E+00  0.000000E+00  0.000000E+00  0.000000E+00  0.000000E+00  0.000000E+00       Funtag_D
      29  6.099907E+05  5.718545E+05       38136.2       13.1190     0.0508867      0.595104      0.197143      0.228439    0.00957374   -0.00416326    0.00781603       1.02722       Opttag_D
      29       59.4477       2.14410       1.00000  0.000000E+00       1.00000  0.000000E+00      0.888726       54.6665       1.00000       18.9726  0.000000E+00       1.75568  0.000000E+00       1.75568  0.000000E+00  0.000000E+00      -3.51136       59.6917       1.81913        1       Scatag_D
      29       1717.48       5752.81  0.000000E+00  0.000000E+00       2460.54  0.000000E+00       2756.03       5512.59  0.000000E+00       10572.7       54.3638       9309.63 -1.000000E+10 -1.000000E+10 -1.000000E+10 -1.000000E+10 -1.000000E+10  0.000000E+00  0.000000E+00  0.000000E+00  0.000000E+00  0.000000E+00  0.000000E+00  0.000000E+00  0.000000E+00  0.000000E+00  0.000000E+00  0.000000E+00  0.000000E+00       Funtag_D
      30  6.099591E+05  5.718137E+05       38145.4       13.2944     0.0522300      0.617657      0.197117      0.228485    0.00966382   -0.00429742    0.00781106       1.02801       Opttag_D
      30       59.4525       2.14331       1.00000  0.000000E+00       1.00000  0.000000E+00      0.888725       54.6712       1.00000       18.9642  0.000000E+00       1.75492  0.000000E+00       1.75492  0.000000E+00  0.000000E+00      -3.50984       59.6917       1.81913        1       Scatag_D
      30       1715.29       5761.69  0.000000E+00  0.000000E+00       2462.95  0.000000E+00       2757.38       5512.65  0.000000E+00       10575.4       54.0151       9306.04 -1.000000E+10 -1.000000E+10 -1.000000E+10 -1.000000E+10 -1.000000E+10  0.000000E+00  0.000000E+00  0.000000E+00  0.000000E+00  0.000000E+00  0.000000E+00  0.000000E+00  0.000000E+00  0.000000E+00  0.000000E+00  0.000000E+00  0.000000E+00       Funtag_D
      31  6.099320E+05  5.717956E+05       38136.4       10.9555     0.0527270      0.626718      0.197104      0.228497    0.00970384   -0.00433310    0.00780490       1.02826       Opttag_D
      31       59.4541       2.14290       1.00000  0.000000E+00       1.00000  0.000000E+00      0.888721       54.6742       1.00000       18.9612  0.000000E+00       1.75445  0.000000E+00       1.75445  0.000000E+00  0.000000E+00      -3.50891       59.6917       1.81913        1       Scatag_D
      31       1712.59       5764.48  0.000000E+00  0.000000E+00       2463.99  0.000000E+00       2756.63       5506.48  0.000000E+00       10575.6       53.6158       9303.01 -1.000000E+10 -1.000000E+10 -1.000000E+10 -1.000000E+10 -1.000000E+10  0.000000E+00  0.000000E+00  0.000000E+00  0.000000E+00  0.000000E+00  0.000000E+00  0.000000E+00  0.000000E+00  0.000000E+00  0.000000E+00  0.000000E+00  0.000000E+00       Funtag_D
      32  6.098798E+05  5.717179E+05       38161.9       13.0724     0.0550287      0.666625      0.197055      0.228556    0.00987524   -0.00448939    0.00781349       1.02967       Opttag_D
      32       59.4615       2.14102       1.00000  0.000000E+00       1.00000  0.000000E+00      0.888710       54.6818       1.00000       18.9503  0.000000E+00       1.75262  0.000000E+00       1.75262  0.000000E+00  0.000000E+00      -3.50523       59.6917       1.81913        1       Scatag_D
      32       1716.36       5780.36  0.000000E+00  0.000000E+00       2468.54  0.000000E+00       2754.76       5513.50  0.000000E+00       10579.8       52.6853       9295.91 -1.000000E+10 -1.000000E+10 -1.000000E+10 -1.000000E+10 -1.000000E+10  0.000000E+00  0.000000E+00  0.000000E+00  0.000000E+00  0.000000E+00  0.000000E+00  0.000000E+00  0.000000E+00  0.000000E+00  0.000000E+00  0.000000E+00  0.000000E+00       Funtag_D
      33  6.098526E+05  5.716634E+05       38189.1       14.9514     0.0568498      0.696602      0.197024      0.228619    0.00999544   -0.00464221    0.00782487       1.03070       Opttag_D
      33       59.4671       2.13969       1.00000  0.000000E+00       1.00000  0.000000E+00      0.888707       54.6838       1.00000       18.9452  0.000000E+00       1.75109  0.000000E+00       1.75109  0.000000E+00  0.000000E+00      -3.50218       59.6917       1.81913        1       Scatag_D
      33       1721.37       5791.92  0.000000E+00  0.000000E+00       2471.85  0.000000E+00       2754.87       5521.41  0.000000E+00       10584.2       52.8043       9290.73 -1.000000E+10 -1.000000E+10 -1.000000E+10 -1.000000E+10 -1.000000E+10  0.000000E+00  0.000000E+00  0.000000E+00  0.000000E+00  0.000000E+00  0.000000E+00  0.000000E+00  0.000000E+00  0.000000E+00  0.000000E+00  0.000000E+00  0.000000E+00       Funtag_D
      34  6.098280E+05  5.716477E+05       38180.3       10.7756     0.0573232      0.704329      0.197017      0.228633     0.0100301   -0.00467293    0.00782622       1.03074       Opttag_D
      34       59.4683       2.13923       1.00000  0.000000E+00       1.00000  0.000000E+00      0.888707       54.6826       1.00000       18.9451  0.000000E+00       1.75066  0.000000E+00       1.75066  0.000000E+00  0.000000E+00      -3.50132       59.6917       1.81913        1       Scatag_D
      34       1721.96       5792.30  0.000000E+00  0.000000E+00       2472.68  0.000000E+00       2754.69       5512.96  0.000000E+00       10585.1       53.0433       9287.49 -1.000000E+10 -1.000000E+10 -1.000000E+10 -1.000000E+10 -1.000000E+10  0.000000E+00  0.000000E+00  0.000000E+00  0.000000E+00  0.000000E+00  0.000000E+00  0.000000E+00  0.000000E+00  0.000000E+00  0.000000E+00  0.000000E+00  0.000000E+00       Funtag_D
      35  6.097824E+05  5.715831E+05       38199.2       9.23369     0.0596991      0.742392      0.196989      0.228709     0.0101727   -0.00486008    0.00784455       1.03157       Opttag_D
      35       59.4746       2.13747       1.00000  0.000000E+00       1.00000  0.000000E+00      0.888703       54.6808       1.00000       18.9441  0.000000E+00       1.74883  0.000000E+00       1.74883  0.000000E+00  0.000000E+00      -3.49767       59.6917       1.81913        1       Scatag_D
      35       1730.04       5801.63  0.000000E+00  0.000000E+00       2476.91  0.000000E+00       2755.57       5514.34  0.000000E+00       10591.0       53.7906       9275.96 -1.000000E+10 -1.000000E+10 -1.000000E+10 -1.000000E+10 -1.000000E+10  0.000000E+00  0.000000E+00  0.000000E+00  0.000000E+00  0.000000E+00  0.000000E+00  0.000000E+00  0.000000E+00  0.000000E+00  0.000000E+00  0.000000E+00  0.000000E+00       Funtag_D
      36  6.097559E+05  5.715481E+05       38207.9       10.4201     0.0614916      0.770699      0.196974      0.228775     0.0102501   -0.00500959    0.00784809       1.03230       Opttag_D
      36       59.4794       2.13666       1.00000  0.000000E+00       1.00000  0.000000E+00      0.888696       54.6848       1.00000       18.9439  0.000000E+00       1.74752  0.000000E+00       1.74752  0.000000E+00  0.000000E+00      -3.49505       59.6917       1.81913        1       Scatag_D
      36       1731.60       5809.85  0.000000E+00  0.000000E+00       2480.22  0.000000E+00       2757.56       5513.73  0.000000E+00       10595.3       53.7130       9265.91 -1.000000E+10 -1.000000E+10 -1.000000E+10 -1.000000E+10 -1.000000E+10  0.000000E+00  0.000000E+00  0.000000E+00  0.000000E+00  0.000000E+00  0.000000E+00  0.000000E+00  0.000000E+00  0.000000E+00  0.000000E+00  0.000000E+00  0.000000E+00       Funtag_D
      37  6.097056E+05  5.714840E+05       38221.6       12.3165     0.0661405      0.841891      0.196955      0.228974     0.0103915   -0.00540994    0.00785912       1.03415       Opttag_D
      37       59.4918       2.13535       1.00000  0.000000E+00       1.00000  0.000000E+00      0.888672       54.6999       1.00000       18.9487  0.000000E+00       1.74435  0.000000E+00       1.74435  0.000000E+00  0.000000E+00      -3.48870       59.6917       1.81913        1       Scatag_D
      37       1736.47       5830.77  0.000000E+00  0.000000E+00       2488.83  0.000000E+00       2765.04       5506.09  0.000000E+00       10607.7       53.2985       9233.47 -1.000000E+10 -1.000000E+10 -1.000000E+10 -1.000000E+10 -1.000000E+10  0.000000E+00  0.000000E+00  0.000000E+00  0.000000E+00  0.000000E+00  0.000000E+00  0.000000E+00  0.000000E+00  0.000000E+00  0.000000E+00  0.000000E+00  0.000000E+00       Funtag_D
      38  6.096694E+05  5.714578E+05       38211.6       16.7310     0.0689583      0.883443      0.196952      0.229099     0.0104492   -0.00562570    0.00785932       1.03477       Opttag_D
      38       59.4994       2.13489       1.00000  0.000000E+00       1.00000  0.000000E+00      0.888643       54.7141       1.00000       18.9568  0.000000E+00       1.74215  0.000000E+00       1.74215  0.000000E+00  0.000000E+00      -3.48431       59.6917       1.81913        1       Scatag_D
      38       1736.56       5837.73  0.000000E+00  0.000000E+00       2494.20  0.000000E+00       2770.87       5504.78  0.000000E+00       10614.5       52.9391       9200.05 -1.000000E+10 -1.000000E+10 -1.000000E+10 -1.000000E+10 -1.000000E+10  0.000000E+00  0.000000E+00  0.000000E+00  0.000000E+00  0.000000E+00  0.000000E+00  0.000000E+00  0.000000E+00  0.000000E+00  0.000000E+00  0.000000E+00  0.000000E+00       Funtag_D
      39  6.096260E+05  5.714554E+05       38170.6       12.1126     0.0694089      0.889725      0.196955      0.229124     0.0104545   -0.00563278    0.00784945       1.03443       Opttag_D
      39       59.5004       2.13484       1.00000  0.000000E+00       1.00000  0.000000E+00      0.888628       54.7201       1.00000       18.9599  0.000000E+00       1.74134  0.000000E+00       1.74134  0.000000E+00  0.000000E+00      -3.48267       59.6917       1.81913        1       Scatag_D
      39       1732.20       5833.89  0.000000E+00  0.000000E+00       2495.22  0.000000E+00       2772.28       5485.59  0.000000E+00       10614.0       52.7433       9184.75 -1.000000E+10 -1.000000E+10 -1.000000E+10 -1.000000E+10 -1.000000E+10  0.000000E+00  0.000000E+00  0.000000E+00  0.000000E+00  0.000000E+00  0.000000E+00  0.000000E+00  0.000000E+00  0.000000E+00  0.000000E+00  0.000000E+00  0.000000E+00       Funtag_D
      40  6.095626E+05  5.714444E+05       38118.2       11.4601     0.0704731      0.924909      0.196960      0.229182     0.0104788   -0.00566454    0.00784543       1.03383       Opttag_D
      40       59.5031       2.13449       1.00000  0.000000E+00       1.00000  0.000000E+00      0.888588       54.7343       1.00000       18.9691  0.000000E+00       1.73970  0.000000E+00       1.73970  0.000000E+00  0.000000E+00      -3.47941       59.6917       1.81913        1       Scatag_D
      40       1730.42       5827.08  0.000000E+00  0.000000E+00       2497.64  0.000000E+00       2775.60       5471.11  0.000000E+00       10613.7       52.7845       9149.84 -1.000000E+10 -1.000000E+10 -1.000000E+10 -1.000000E+10 -1.000000E+10  0.000000E+00  0.000000E+00  0.000000E+00  0.000000E+00  0.000000E+00  0.000000E+00  0.000000E+00  0.000000E+00  0.000000E+00  0.000000E+00  0.000000E+00  0.000000E+00       Funtag_D
      41  6.095258E+05  5.714254E+05       38100.4       14.4175     0.0712466      0.971928      0.196956      0.229229     0.0105207   -0.00572688    0.00784823       1.03328       Opttag_D
      41       59.5052       2.13380       1.00000  0.000000E+00       1.00000  0.000000E+00      0.888557       54.7443       1.00000       18.9775  0.000000E+00       1.73890  0.000000E+00       1.73890  0.000000E+00  0.000000E+00      -3.47779       59.6917       1.81913        1       Scatag_D
      41       1731.66       5820.89  0.000000E+00  0.000000E+00       2499.42  0.000000E+00       2776.61       5475.86  0.000000E+00       10614.6       53.1790       9128.15 -1.000000E+10 -1.000000E+10 -1.000000E+10 -1.000000E+10 -1.000000E+10  0.000000E+00  0.000000E+00  0.000000E+00  0.000000E+00  0.000000E+00  0.000000E+00  0.000000E+00  0.000000E+00  0.000000E+00  0.000000E+00  0.000000E+00  0.000000E+00       Funtag_D
      42  6.094811E+05  5.714099E+05       38071.2       10.4230     0.0707769      0.989998      0.196949      0.229233     0.0105549   -0.00572221    0.00784888       1.03233       Opttag_D
      42       59.5042       2.13312       1.00000  0.000000E+00       1.00000  0.000000E+00      0.888535       54.7492       1.00000       18.9820  0.000000E+00       1.73933  0.000000E+00       1.73933  0.000000E+00  0.000000E+00      -3.47866       59.6917       1.81913        1       Scatag_D
      42       1731.95       5810.24  0.000000E+00  0.000000E+00       2498.93  0.000000E+00       2774.28       5471.59  0.000000E+00       10612.8       53.8041       9117.66 -1.000000E+10 -1.000000E+10 -1.000000E+10 -1.000000E+10 -1.000000E+10  0.000000E+00  0.000000E+00  0.000000E+00  0.000000E+00  0.000000E+00  0.000000E+00  0.000000E+00  0.000000E+00  0.000000E+00  0.000000E+00  0.000000E+00  0.000000E+00       Funtag_D
      43  6.094469E+05  5.713929E+05       38054.1       9.58357     0.0705896       1.00396      0.196941      0.229242     0.0105925   -0.00576602    0.00784667       1.03157       Opttag_D
      43       59.5044       2.13253       1.00000  0.000000E+00       1.00000  0.000000E+00      0.888521       54.7560       1.00000       18.9856  0.000000E+00       1.74013  0.000000E+00       1.74013  0.000000E+00  0.000000E+00      -3.48026       59.6917       1.81913        1       Scatag_D
      43       1730.97       5801.67  0.000000E+00  0.000000E+00       2498.75  0.000000E+00       2771.73       5475.02  0.000000E+00       10613.5       54.0554       9108.38 -1.000000E+10 -1.000000E+10 -1.000000E+10 -1.000000E+10 -1.000000E+10  0.000000E+00  0.000000E+00  0.000000E+00  0.000000E+00  0.000000E+00  0.000000E+00  0.000000E+00  0.000000E+00  0.000000E+00  0.000000E+00  0.000000E+00  0.000000E+00       Funtag_D
      44  6.094212E+05  5.713771E+05       38044.1       10.6084     0.0705030       1.01671      0.196933      0.229249     0.0106274   -0.00582098    0.00784758       1.03089       Opttag_D
      44       59.5050       2.13211       1.00000  0.000000E+00       1.00000  0.000000E+00      0.888507       54.7665       1.00000       18.9887  0.000000E+00       1.74100  0.000000E+00       1.74100  0.000000E+00  0.000000E+00      -3.48200       59.6917       1.81913        1       Scatag_D
      44       1731.37       5794.08  0.000000E+00  0.000000E+00       2498.70  0.000000E+00       2769.69       5481.14  0.000000E+00       10615.5       54.0009       9099.62 -1.000000E+10 -1.000000E+10 -1.000000E+10 -1.000000E+10 -1.000000E+10  0.000000E+00  0.000000E+00  0.000000E+00  0.000000E+00  0.000000E+00  0.000000E+00  0.000000E+00  0.000000E+00  0.000000E+00  0.000000E+00  0.000000E+00  0.000000E+00       Funtag_D
      45  6.093988E+05  5.713717E+05       38027.1       8.37927     0.0707745       1.02589      0.196929      0.229257     0.0106391   -0.00585365    0.00784511       1.03061       Opttag_D
      45       59.5060       2.13207       1.00000  0.000000E+00       1.00000  0.000000E+00      0.888498       54.7754       1.00000       18.9904  0.000000E+00       1.74128  0.000000E+00       1.74128  0.000000E+00  0.000000E+00      -3.48255       59.6917       1.81913        1       Scatag_D
      45       1730.28       5790.88  0.000000E+00  0.000000E+00       2499.17  0.000000E+00       2769.30       5473.37  0.000000E+00       10617.6       53.6956       9092.84 -1.000000E+10 -1.000000E+10 -1.000000E+10 -1.000000E+10 -1.000000E+10  0.000000E+00  0.000000E+00  0.000000E+00  0.000000E+00  0.000000E+00  0.000000E+00  0.000000E+00  0.000000E+00  0.000000E+00  0.000000E+00  0.000000E+00  0.000000E+00       Funtag_D
      46  6.093674E+05  5.713643E+05       38003.1       8.72446     0.0715250       1.04226      0.196927      0.229271     0.0106555   -0.00591087    0.00784472       1.03026       Opttag_D
      46       59.5083       2.13214       1.00000  0.000000E+00       1.00000  0.000000E+00      0.888478       54.7941       1.00000       18.9934  0.000000E+00       1.74162  0.000000E+00       1.74162  0.000000E+00  0.000000E+00      -3.48325       59.6917       1.81913        1       Scatag_D
      46       1730.11       5786.97  0.000000E+00  0.000000E+00       2500.42  0.000000E+00       2769.19       5462.71  0.000000E+00       10622.0       53.0267       9078.65 -1.000000E+10 -1.000000E+10 -1.000000E+10 -1.000000E+10 -1.000000E+10  0.000000E+00  0.000000E+00  0.000000E+00  0.000000E+00  0.000000E+00  0.000000E+00  0.000000E+00  0.000000E+00  0.000000E+00  0.000000E+00  0.000000E+00  0.000000E+00       Funtag_D
      47  6.093415E+05  5.713541E+05       37987.4       13.1370     0.0722440       1.05461      0.196919      0.229293     0.0106781   -0.00598043    0.00784904       1.02981       Opttag_D
      47       59.5096       2.13204       1.00000  0.000000E+00       1.00000  0.000000E+00      0.888450       54.8131       1.00000       18.9977  0.000000E+00       1.74173  0.000000E+00       1.74173  0.000000E+00  0.000000E+00      -3.48346       59.6917       1.81913        1       Scatag_D
      47       1732.02       5781.93  0.000000E+00  0.000000E+00       2501.61  0.000000E+00       2768.88       5463.20  0.000000E+00       10626.2       52.6976       9060.85 -1.000000E+10 -1.000000E+10 -1.000000E+10 -1.000000E+10 -1.000000E+10  0.000000E+00  0.000000E+00  0.000000E+00  0.000000E+00  0.000000E+00  0.000000E+00  0.000000E+00  0.000000E+00  0.000000E+00  0.000000E+00  0.000000E+00  0.000000E+00       Funtag_D
      48  6.093092E+05  5.713470E+05       37962.2       9.91128     0.0727611       1.05861      0.196913      0.229309     0.0106936   -0.00603057    0.00785218       1.02945       Opttag_D
      48       59.5102       2.13196       1.00000  0.000000E+00       1.00000  0.000000E+00      0.888427       54.8263       1.00000       19.0011  0.000000E+00       1.74192  0.000000E+00       1.74192  0.000000E+00  0.000000E+00      -3.48384       59.6917       1.81913        1       Scatag_D
      48       1733.40       5777.86  0.000000E+00  0.000000E+00       2502.46  0.000000E+00       2768.55       5451.75  0.000000E+00       10629.2       52.6266       9046.32 -1.000000E+10 -1.000000E+10 -1.000000E+10 -1.000000E+10 -1.000000E+10  0.000000E+00  0.000000E+00  0.000000E+00  0.000000E+00  0.000000E+00  0.000000E+00  0.000000E+00  0.000000E+00  0.000000E+00  0.000000E+00  0.000000E+00  0.000000E+00       Funtag_D
      49  6.092735E+05  5.713344E+05       37939.1       9.62441     0.0733186       1.05288      0.196903      0.229337     0.0107216   -0.00610796    0.00785443       1.02889       Opttag_D
      49       59.5096       2.13159       1.00000  0.000000E+00       1.00000  0.000000E+00      0.888393       54.8388       1.00000       19.0071  0.000000E+00       1.74195  0.000000E+00       1.74195  0.000000E+00  0.000000E+00      -3.48390       59.6917       1.81913        1       Scatag_D
      49       1734.39       5771.56  0.000000E+00  0.000000E+00       2503.44  0.000000E+00       2767.81       5450.63  0.000000E+00       10632.1       52.8425       9026.38 -1.000000E+10 -1.000000E+10 -1.000000E+10 -1.000000E+10 -1.000000E+10  0.000000E+00  0.000000E+00  0.000000E+00  0.000000E+00  0.000000E+00  0.000000E+00  0.000000E+00  0.000000E+00  0.000000E+00  0.000000E+00  0.000000E+00  0.000000E+00       Funtag_D
      50  6.092535E+05  5.713264E+05       37927.1       10.1342     0.0734600       1.04163      0.196894      0.229357     0.0107391   -0.00614966    0.00785010       1.02855       Opttag_D
      50       59.5083       2.13130       1.00000  0.000000E+00       1.00000  0.000000E+00      0.888378       54.8409       1.00000       19.0098  0.000000E+00       1.74154  0.000000E+00       1.74154  0.000000E+00  0.000000E+00      -3.48308       59.6917       1.81913        1       Scatag_D
      50       1732.48       5767.73  0.000000E+00  0.000000E+00       2503.82  0.000000E+00       2767.12       5454.06  0.000000E+00       10631.6       52.9399       9017.28 -1.000000E+10 -1.000000E+10 -1.000000E+10 -1.000000E+10 -1.000000E+10  0.000000E+00  0.000000E+00  0.000000E+00  0.000000E+00  0.000000E+00  0.000000E+00  0.000000E+00  0.000000E+00  0.000000E+00  0.000000E+00  0.000000E+00  0.000000E+00       Funtag_D
      51  6.092361E+05  5.713198E+05       37916.3       7.35218     0.0734589       1.03284      0.196889      0.229363     0.0107537   -0.00615644    0.00784427       1.02838       Opttag_D
      51       59.5073       2.13102       1.00000  0.000000E+00       1.00000  0.000000E+00      0.888373       54.8387       1.00000       19.0111  0.000000E+00       1.74119  0.000000E+00       1.74119  0.000000E+00  0.000000E+00      -3.48239       59.6917       1.81913        1       Scatag_D
      51       1729.91       5765.87  0.000000E+00  0.000000E+00       2503.96  0.000000E+00       2766.58       5453.32  0.000000E+00       10630.3       52.9306       9013.43 -1.000000E+10 -1.000000E+10 -1.000000E+10 -1.000000E+10 -1.000000E+10  0.000000E+00  0.000000E+00  0.000000E+00  0.000000E+00  0.000000E+00  0.000000E+00  0.000000E+00  0.000000E+00  0.000000E+00  0.000000E+00  0.000000E+00  0.000000E+00       Funtag_D
      52  6.092162E+05  5.713072E+05       37909.1       7.16223     0.0735507       1.01777      0.196883      0.229366     0.0107815   -0.00614605    0.00783582       1.02822       Opttag_D
      52       59.5057       2.13053       1.00000  0.000000E+00       1.00000  0.000000E+00      0.888365       54.8365       1.00000       19.0137  0.000000E+00       1.74032  0.000000E+00       1.74032  0.000000E+00  0.000000E+00      -3.48064       59.6917       1.81913        1       Scatag_D
      52       1726.19       5764.03  0.000000E+00  0.000000E+00       2504.42  0.000000E+00       2765.97       5458.33  0.000000E+00       10627.9       52.7332       9009.45 -1.000000E+10 -1.000000E+10 -1.000000E+10 -1.000000E+10 -1.000000E+10  0.000000E+00  0.000000E+00  0.000000E+00  0.000000E+00  0.000000E+00  0.000000E+00  0.000000E+00  0.000000E+00  0.000000E+00  0.000000E+00  0.000000E+00  0.000000E+00       Funtag_D
      53  6.092041E+05  5.712988E+05       37905.3       7.75308     0.0737625       1.01159      0.196875      0.229357     0.0108001   -0.00611835    0.00783306       1.02811       Opttag_D
      53       59.5051       2.13027       1.00000  0.000000E+00       1.00000  0.000000E+00      0.888358       54.8384       1.00000       19.0159  0.000000E+00       1.73987  0.000000E+00       1.73987  0.000000E+00  0.000000E+00      -3.47973       59.6917       1.81913        1       Scatag_D
      53       1724.97       5762.81  0.000000E+00  0.000000E+00       2504.94  0.000000E+00       2765.69       5459.73  0.000000E+00       10626.7       52.5467       9007.91 -1.000000E+10 -1.000000E+10 -1.000000E+10 -1.000000E+10 -1.000000E+10  0.000000E+00  0.000000E+00  0.000000E+00  0.000000E+00  0.000000E+00  0.000000E+00  0.000000E+00  0.000000E+00  0.000000E+00  0.000000E+00  0.000000E+00  0.000000E+00       Funtag_D
      54  6.091913E+05  5.712914E+05       37899.9       6.54794     0.0739925       1.00842      0.196871      0.229343     0.0108163   -0.00607333    0.00783318       1.02801       Opttag_D
      54       59.5048       2.13004       1.00000  0.000000E+00       1.00000  0.000000E+00      0.888352       54.8405       1.00000       19.0180  0.000000E+00       1.73947  0.000000E+00       1.73947  0.000000E+00  0.000000E+00      -3.47894       59.6917       1.81913        1       Scatag_D
      54       1725.02       5761.66  0.000000E+00  0.000000E+00       2505.47  0.000000E+00       2765.32       5457.67  0.000000E+00       10625.4       52.3847       9007.03 -1.000000E+10 -1.000000E+10 -1.000000E+10 -1.000000E+10 -1.000000E+10  0.000000E+00  0.000000E+00  0.000000E+00  0.000000E+00  0.000000E+00  0.000000E+00  0.000000E+00  0.000000E+00  0.000000E+00  0.000000E+00  0.000000E+00  0.000000E+00       Funtag_D
      55  6.091753E+05  5.712717E+05       37903.6       8.37656     0.0746266       1.00590      0.196857      0.229311     0.0108599   -0.00596763    0.00784383       1.02791       Opttag_D
      55       59.5045       2.12937       1.00000  0.000000E+00       1.00000  0.000000E+00      0.888337       54.8454       1.00000       19.0240  0.000000E+00       1.73852  0.000000E+00       1.73852  0.000000E+00  0.000000E+00      -3.47705       59.6917       1.81913        1       Scatag_D
      55       1729.72       5760.54  0.000000E+00  0.000000E+00       2506.84  0.000000E+00       2763.91       5462.32  0.000000E+00       10621.6       52.1724       9006.52 -1.000000E+10 -1.000000E+10 -1.000000E+10 -1.000000E+10 -1.000000E+10  0.000000E+00  0.000000E+00  0.000000E+00  0.000000E+00  0.000000E+00  0.000000E+00  0.000000E+00  0.000000E+00  0.000000E+00  0.000000E+00  0.000000E+00  0.000000E+00       Funtag_D
      56  6.091628E+05  5.712640E+05       37898.8       6.51601     0.0748815       1.00643      0.196852      0.229300     0.0108769   -0.00592412    0.00784738       1.02788       Opttag_D
      56       59.5046       2.12908       1.00000  0.000000E+00       1.00000  0.000000E+00      0.888331       54.8461       1.00000       19.0269  0.000000E+00       1.73824  0.000000E+00       1.73824  0.000000E+00  0.000000E+00      -3.47648       59.6917       1.81913        1       Scatag_D
      56       1731.28       5760.26  0.000000E+00  0.000000E+00       2507.43  0.000000E+00       2762.40       5462.05  0.000000E+00       10618.6       52.1762       9004.68 -1.000000E+10 -1.000000E+10 -1.000000E+10 -1.000000E+10 -1.000000E+10  0.000000E+00  0.000000E+00  0.000000E+00  0.000000E+00  0.000000E+00  0.000000E+00  0.000000E+00  0.000000E+00  0.000000E+00  0.000000E+00  0.000000E+00  0.000000E+00       Funtag_D
      57  6.091541E+05  5.712602E+05       37893.9       5.52108     0.0749611       1.00870      0.196849      0.229300     0.0108852   -0.00591945    0.00784760       1.02786       Opttag_D
      57       59.5048       2.12893       1.00000  0.000000E+00       1.00000  0.000000E+00      0.888327       54.8456       1.00000       19.0282  0.000000E+00       1.73816  0.000000E+00       1.73816  0.000000E+00  0.000000E+00      -3.47633       59.6917       1.81913        1       Scatag_D
      57       1731.38       5760.05  0.000000E+00  0.000000E+00       2507.63  0.000000E+00       2761.13       5463.37  0.000000E+00       10616.4       52.2287       9001.77 -1.000000E+10 -1.000000E+10 -1.000000E+10 -1.000000E+10 -1.000000E+10  0.000000E+00  0.000000E+00  0.000000E+00  0.000000E+00  0.000000E+00  0.000000E+00  0.000000E+00  0.000000E+00  0.000000E+00  0.000000E+00  0.000000E+00  0.000000E+00       Funtag_D
      58  6.091361E+05  5.712505E+05       37885.6       6.74860     0.0754141       1.01704      0.196841      0.229301     0.0109067   -0.00590214    0.00785192       1.02802       Opttag_D
      58       59.5061       2.12856       1.00000  0.000000E+00       1.00000  0.000000E+00      0.888311       54.8475       1.00000       19.0343  0.000000E+00       1.73760  0.000000E+00       1.73760  0.000000E+00  0.000000E+00      -3.47519       59.6917       1.81913        1       Scatag_D
      58       1733.29       5761.80  0.000000E+00  0.000000E+00       2508.74  0.000000E+00       2756.49       5472.41  0.000000E+00       10607.4       52.2350       8993.27 -1.000000E+10 -1.000000E+10 -1.000000E+10 -1.000000E+10 -1.000000E+10  0.000000E+00  0.000000E+00  0.000000E+00  0.000000E+00  0.000000E+00  0.000000E+00  0.000000E+00  0.000000E+00  0.000000E+00  0.000000E+00  0.000000E+00  0.000000E+00       Funtag_D
      59  6.091285E+05  5.712488E+05       37879.8       11.1823     0.0760873       1.02331      0.196839      0.229305     0.0109105   -0.00588512    0.00785276       1.02819       Opttag_D
      59       59.5081       2.12858       1.00000  0.000000E+00       1.00000  0.000000E+00      0.888293       54.8549       1.00000       19.0416  0.000000E+00       1.73706  0.000000E+00       1.73706  0.000000E+00  0.000000E+00      -3.47412       59.6917       1.81913        1       Scatag_D
      59       1733.66       5763.76  0.000000E+00  0.000000E+00       2510.19  0.000000E+00       2752.62       5482.00  0.000000E+00       10598.4       52.0072       8987.10 -1.000000E+10 -1.000000E+10 -1.000000E+10 -1.000000E+10 -1.000000E+10  0.000000E+00  0.000000E+00  0.000000E+00  0.000000E+00  0.000000E+00  0.000000E+00  0.000000E+00  0.000000E+00  0.000000E+00  0.000000E+00  0.000000E+00  0.000000E+00       Funtag_D
      60  6.091162E+05  5.712517E+05       37864.4       6.14221     0.0761168       1.02371      0.196842      0.229309     0.0109039   -0.00588813    0.00784905       1.02806       Opttag_D
      60       59.5084       2.12871       1.00000  0.000000E+00       1.00000  0.000000E+00      0.888292       54.8562       1.00000       19.0418  0.000000E+00       1.73698  0.000000E+00       1.73698  0.000000E+00  0.000000E+00      -3.47396       59.6917       1.81913        1       Scatag_D
      60       1732.02       5762.25  0.000000E+00  0.000000E+00       2510.24  0.000000E+00       2752.42       5472.51  0.000000E+00       10597.5       51.9485       8985.54 -1.000000E+10 -1.000000E+10 -1.000000E+10 -1.000000E+10 -1.000000E+10  0.000000E+00  0.000000E+00  0.000000E+00  0.000000E+00  0.000000E+00  0.000000E+00  0.000000E+00  0.000000E+00  0.000000E+00  0.000000E+00  0.000000E+00  0.000000E+00       Funtag_D
      61  6.091041E+05  5.712588E+05       37845.4       4.91140     0.0764623       1.02594      0.196846      0.229318     0.0108884   -0.00588587    0.00784465       1.02790       Opttag_D
      61       59.5098       2.12903       1.00000  0.000000E+00       1.00000  0.000000E+00      0.888283       54.8623       1.00000       19.0452  0.000000E+00       1.73667  0.000000E+00       1.73667  0.000000E+00  0.000000E+00      -3.47333       59.6917       1.81913        1       Scatag_D
      61       1730.08       5760.41  0.000000E+00  0.000000E+00       2510.88  0.000000E+00       2750.90       5467.68  0.000000E+00       10592.6       51.7756       8981.00 -1.000000E+10 -1.000000E+10 -1.000000E+10 -1.000000E+10 -1.000000E+10  0.000000E+00  0.000000E+00  0.000000E+00  0.000000E+00  0.000000E+00  0.000000E+00  0.000000E+00  0.000000E+00  0.000000E+00  0.000000E+00  0.000000E+00  0.000000E+00       Funtag_D
      62  6.090943E+05  5.712675E+05       37826.9       5.73500     0.0769716       1.02901      0.196850      0.229329     0.0108692   -0.00588045    0.00784410       1.02775       Opttag_D
      62       59.5119       2.12943       1.00000  0.000000E+00       1.00000  0.000000E+00      0.888271       54.8697       1.00000       19.0505  0.000000E+00       1.73637  0.000000E+00       1.73637  0.000000E+00  0.000000E+00      -3.47274       59.6917       1.81913        1       Scatag_D
      62       1729.84       5758.79  0.000000E+00  0.000000E+00       2511.76  0.000000E+00       2748.82       5465.17  0.000000E+00       10585.6       51.6881       8975.23 -1.000000E+10 -1.000000E+10 -1.000000E+10 -1.000000E+10 -1.000000E+10  0.000000E+00  0.000000E+00  0.000000E+00  0.000000E+00  0.000000E+00  0.000000E+00  0.000000E+00  0.000000E+00  0.000000E+00  0.000000E+00  0.000000E+00  0.000000E+00       Funtag_D
      63  6.090814E+05  5.712895E+05       37791.8       9.56286     0.0779721       1.03233      0.196864      0.229358     0.0108205   -0.00589521    0.00784609       1.02722       Opttag_D
      63       59.5162       2.13037       1.00000  0.000000E+00       1.00000  0.000000E+00      0.888249       54.8838       1.00000       19.0606  0.000000E+00       1.73600  0.000000E+00       1.73600  0.000000E+00  0.000000E+00      -3.47199       59.6917       1.81913        1       Scatag_D
      63       1730.71       5752.82  0.000000E+00  0.000000E+00       2513.21  0.000000E+00       2745.74       5462.50  0.000000E+00       10571.5       51.7303       8963.65 -1.000000E+10 -1.000000E+10 -1.000000E+10 -1.000000E+10 -1.000000E+10  0.000000E+00  0.000000E+00  0.000000E+00  0.000000E+00  0.000000E+00  0.000000E+00  0.000000E+00  0.000000E+00  0.000000E+00  0.000000E+00  0.000000E+00  0.000000E+00       Funtag_D
      64  6.090658E+05  5.713103E+05       37755.5       6.78164     0.0787328       1.03368      0.196879      0.229379     0.0107747   -0.00590259    0.00784873       1.02669       Opttag_D
      64       59.5195       2.13121       1.00000  0.000000E+00       1.00000  0.000000E+00      0.888233       54.8942       1.00000       19.0681  0.000000E+00       1.73601  0.000000E+00       1.73601  0.000000E+00  0.000000E+00      -3.47201       59.6917       1.81913        1       Scatag_D
      64       1731.88       5746.95  0.000000E+00  0.000000E+00       2514.14  0.000000E+00       2743.85       5451.22  0.000000E+00       10560.8       51.8593       8954.84 -1.000000E+10 -1.000000E+10 -1.000000E+10 -1.000000E+10 -1.000000E+10  0.000000E+00  0.000000E+00  0.000000E+00  0.000000E+00  0.000000E+00  0.000000E+00  0.000000E+00  0.000000E+00  0.000000E+00  0.000000E+00  0.000000E+00  0.000000E+00       Funtag_D
      65  6.090543E+05  5.713172E+05       37737.1       5.84857     0.0788918       1.03221      0.196886      0.229388     0.0107595   -0.00592216    0.00784699       1.02627       Opttag_D
      65       59.5202       2.13147       1.00000  0.000000E+00       1.00000  0.000000E+00      0.888228       54.8963       1.00000       19.0692  0.000000E+00       1.73622  0.000000E+00       1.73622  0.000000E+00  0.000000E+00      -3.47244       59.6917       1.81913        1       Scatag_D
      65       1731.11       5742.20  0.000000E+00  0.000000E+00       2514.14  0.000000E+00       2743.74       5446.38  0.000000E+00       10557.2       51.9923       8950.38 -1.000000E+10 -1.000000E+10 -1.000000E+10 -1.000000E+10 -1.000000E+10  0.000000E+00  0.000000E+00  0.000000E+00  0.000000E+00  0.000000E+00  0.000000E+00  0.000000E+00  0.000000E+00  0.000000E+00  0.000000E+00  0.000000E+00  0.000000E+00       Funtag_D
      66  6.090345E+05  5.713435E+05       37690.9       7.20043     0.0795805       1.02964      0.196907      0.229426     0.0107014   -0.00599594    0.00784422       1.02516       Opttag_D
      66       59.5226       2.13242       1.00000  0.000000E+00       1.00000  0.000000E+00      0.888208       54.9051       1.00000       19.0746  0.000000E+00       1.73673  0.000000E+00       1.73673  0.000000E+00  0.000000E+00      -3.47346       59.6917       1.81913        1       Scatag_D
      66       1729.89       5729.75  0.000000E+00  0.000000E+00       2514.48  0.000000E+00       2742.93       5437.04  0.000000E+00       10546.0       52.2206       8938.67 -1.000000E+10 -1.000000E+10 -1.000000E+10 -1.000000E+10 -1.000000E+10  0.000000E+00  0.000000E+00  0.000000E+00  0.000000E+00  0.000000E+00  0.000000E+00  0.000000E+00  0.000000E+00  0.000000E+00  0.000000E+00  0.000000E+00  0.000000E+00       Funtag_D
      67  6.090276E+05  5.713800E+05       37647.6       11.3285     0.0805732       1.03019      0.196934      0.229481     0.0106209   -0.00610209    0.00783649       1.02422       Opttag_D
      67       59.5251       2.13370       1.00000  0.000000E+00       1.00000  0.000000E+00      0.888182       54.9186       1.00000       19.0825  0.000000E+00       1.73710  0.000000E+00       1.73710  0.000000E+00  0.000000E+00      -3.47419       59.6917       1.81913        1       Scatag_D
      67       1726.48       5719.24  0.000000E+00  0.000000E+00       2515.29  0.000000E+00       2741.43       5430.42  0.000000E+00       10534.5       52.1447       8928.11 -1.000000E+10 -1.000000E+10 -1.000000E+10 -1.000000E+10 -1.000000E+10  0.000000E+00  0.000000E+00  0.000000E+00  0.000000E+00  0.000000E+00  0.000000E+00  0.000000E+00  0.000000E+00  0.000000E+00  0.000000E+00  0.000000E+00  0.000000E+00       Funtag_D
      68  6.090148E+05  5.713780E+05       37636.8       5.31346     0.0805680       1.03133      0.196933      0.229481     0.0106252   -0.00609998    0.00783274       1.02419       Opttag_D
      68       59.5246       2.13360       1.00000  0.000000E+00       1.00000  0.000000E+00      0.888182       54.9180       1.00000       19.0822  0.000000E+00       1.73706  0.000000E+00       1.73706  0.000000E+00  0.000000E+00      -3.47411       59.6917       1.81913        1       Scatag_D
      68       1724.83       5718.97  0.000000E+00  0.000000E+00       2515.36  0.000000E+00       2741.08       5422.11  0.000000E+00       10535.3       52.0279       8927.02 -1.000000E+10 -1.000000E+10 -1.000000E+10 -1.000000E+10 -1.000000E+10  0.000000E+00  0.000000E+00  0.000000E+00  0.000000E+00  0.000000E+00  0.000000E+00  0.000000E+00  0.000000E+00  0.000000E+00  0.000000E+00  0.000000E+00  0.000000E+00       Funtag_D
      69  6.090063E+05  5.713875E+05       37618.9       4.00336     0.0809437       1.03405      0.196941      0.229501     0.0106044   -0.00612919    0.00782634       1.02396       Opttag_D
      69       59.5248       2.13389       1.00000  0.000000E+00       1.00000  0.000000E+00      0.888174       54.9221       1.00000       19.0850  0.000000E+00       1.73705  0.000000E+00       1.73705  0.000000E+00  0.000000E+00      -3.47409       59.6917       1.81913        1       Scatag_D
      69       1722.01       5716.35  0.000000E+00  0.000000E+00       2515.84  0.000000E+00       2739.97       5417.53  0.000000E+00       10533.0       51.8266       8922.35 -1.000000E+10 -1.000000E+10 -1.000000E+10 -1.000000E+10 -1.000000E+10  0.000000E+00  0.000000E+00  0.000000E+00  0.000000E+00  0.000000E+00  0.000000E+00  0.000000E+00  0.000000E+00  0.000000E+00  0.000000E+00  0.000000E+00  0.000000E+00       Funtag_D
      70  6.089989E+05  5.714029E+05       37596.0       4.70552     0.0815646       1.03772      0.196953      0.229532     0.0105704   -0.00617436    0.00782084       1.02361       Opttag_D
      70       59.5253       2.13435       1.00000  0.000000E+00       1.00000  0.000000E+00      0.888160       54.9285       1.00000       19.0896  0.000000E+00       1.73715  0.000000E+00       1.73715  0.000000E+00  0.000000E+00      -3.47429       59.6917       1.81913        1       Scatag_D
      70       1719.59       5712.50  0.000000E+00  0.000000E+00       2516.51  0.000000E+00       2738.66       5412.28  0.000000E+00       10528.9       51.6675       8915.90 -1.000000E+10 -1.000000E+10 -1.000000E+10 -1.000000E+10 -1.000000E+10  0.000000E+00  0.000000E+00  0.000000E+00  0.000000E+00  0.000000E+00  0.000000E+00  0.000000E+00  0.000000E+00  0.000000E+00  0.000000E+00  0.000000E+00  0.000000E+00       Funtag_D
      71  6.089864E+05  5.714213E+05       37565.1       7.55515     0.0828170       1.06318      0.196970      0.229588     0.0105297   -0.00624196    0.00781626       1.02312       Opttag_D
      71       59.5257       2.13480       1.00000  0.000000E+00       1.00000  0.000000E+00      0.888137       54.9378       1.00000       19.0984  0.000000E+00       1.73733  0.000000E+00       1.73733  0.000000E+00  0.000000E+00      -3.47466       59.6917       1.81913        1       Scatag_D
      71       1717.58       5707.01  0.000000E+00  0.000000E+00       2517.71  0.000000E+00       2736.90       5407.89  0.000000E+00       10522.1       51.6242       8904.26 -1.000000E+10 -1.000000E+10 -1.000000E+10 -1.000000E+10 -1.000000E+10  0.000000E+00  0.000000E+00  0.000000E+00  0.000000E+00  0.000000E+00  0.000000E+00  0.000000E+00  0.000000E+00  0.000000E+00  0.000000E+00  0.000000E+00  0.000000E+00       Funtag_D
      72  6.089735E+05  5.714473E+05       37526.3       7.29331     0.0846661       1.11536      0.196994      0.229663     0.0104724   -0.00631137    0.00781621       1.02259       Opttag_D
      72       59.5264       2.13542       1.00000  0.000000E+00       1.00000  0.000000E+00      0.888106       54.9503       1.00000       19.1112  0.000000E+00       1.73790  0.000000E+00       1.73790  0.000000E+00  0.000000E+00      -3.47581       59.6917       1.81913        1       Scatag_D
      72       1717.56       5701.13  0.000000E+00  0.000000E+00       2519.29  0.000000E+00       2735.54       5397.29  0.000000E+00       10513.0       51.6586       8890.75 -1.000000E+10 -1.000000E+10 -1.000000E+10 -1.000000E+10 -1.000000E+10  0.000000E+00  0.000000E+00  0.000000E+00  0.000000E+00  0.000000E+00  0.000000E+00  0.000000E+00  0.000000E+00  0.000000E+00  0.000000E+00  0.000000E+00  0.000000E+00       Funtag_D
      73  6.089623E+05  5.714398E+05       37522.5       5.22776     0.0850368       1.12312      0.196991      0.229672     0.0104889   -0.00630595    0.00781794       1.02259       Opttag_D
      73       59.5263       2.13512       1.00000  0.000000E+00       1.00000  0.000000E+00      0.888103       54.9501       1.00000       19.1129  0.000000E+00       1.73794  0.000000E+00       1.73794  0.000000E+00  0.000000E+00      -3.47588       59.6917       1.81913        1       Scatag_D
      73       1718.32       5701.08  0.000000E+00  0.000000E+00       2519.47  0.000000E+00       2736.20       5396.45  0.000000E+00       10511.8       51.7746       8887.36 -1.000000E+10 -1.000000E+10 -1.000000E+10 -1.000000E+10 -1.000000E+10  0.000000E+00  0.000000E+00  0.000000E+00  0.000000E+00  0.000000E+00  0.000000E+00  0.000000E+00  0.000000E+00  0.000000E+00  0.000000E+00  0.000000E+00  0.000000E+00       Funtag_D
      74  6.089444E+05  5.714193E+05       37525.2       6.23440     0.0860739       1.14407      0.196984      0.229700     0.0105343   -0.00628698    0.00782787       1.02270       Opttag_D
      74       59.5260       2.13430       1.00000  0.000000E+00       1.00000  0.000000E+00      0.888092       54.9499       1.00000       19.1177  0.000000E+00       1.73807  0.000000E+00       1.73807  0.000000E+00  0.000000E+00      -3.47614       59.6917       1.81913        1       Scatag_D
      74       1722.68       5702.33  0.000000E+00  0.000000E+00       2519.94  0.000000E+00       2738.58       5402.14  0.000000E+00       10508.5       52.0337       8878.93 -1.000000E+10 -1.000000E+10 -1.000000E+10 -1.000000E+10 -1.000000E+10  0.000000E+00  0.000000E+00  0.000000E+00  0.000000E+00  0.000000E+00  0.000000E+00  0.000000E+00  0.000000E+00  0.000000E+00  0.000000E+00  0.000000E+00  0.000000E+00       Funtag_D
      75  6.089301E+05  5.714088E+05       37521.3       8.42771     0.0876707       1.19740      0.196986      0.229757     0.0105574   -0.00631288    0.00783797       1.02274       Opttag_D
      75       59.5263       2.13380       1.00000  0.000000E+00       1.00000  0.000000E+00      0.888074       54.9539       1.00000       19.1265  0.000000E+00       1.73838  0.000000E+00       1.73838  0.000000E+00  0.000000E+00      -3.47676       59.6917       1.81913        1       Scatag_D
      75       1727.13       5702.75  0.000000E+00  0.000000E+00       2520.83  0.000000E+00       2741.28       5407.90  0.000000E+00       10503.3       52.0845       8866.06 -1.000000E+10 -1.000000E+10 -1.000000E+10 -1.000000E+10 -1.000000E+10  0.000000E+00  0.000000E+00  0.000000E+00  0.000000E+00  0.000000E+00  0.000000E+00  0.000000E+00  0.000000E+00  0.000000E+00  0.000000E+00  0.000000E+00  0.000000E+00       Funtag_D
      76  6.089073E+05  5.714037E+05       37503.6       7.45135     0.0900071       1.27798      0.196991      0.229850     0.0105686   -0.00639072    0.00784893       1.02276       Opttag_D
      76       59.5270       2.13345       1.00000  0.000000E+00       1.00000  0.000000E+00      0.888047       54.9622       1.00000       19.1401  0.000000E+00       1.73879  0.000000E+00       1.73879  0.000000E+00  0.000000E+00      -3.47758       59.6917       1.81913        1       Scatag_D
      76       1731.97       5702.98  0.000000E+00  0.000000E+00       2522.16  0.000000E+00       2745.16       5409.06  0.000000E+00       10496.4       51.9578       8843.96 -1.000000E+10 -1.000000E+10 -1.000000E+10 -1.000000E+10 -1.000000E+10  0.000000E+00  0.000000E+00  0.000000E+00  0.000000E+00  0.000000E+00  0.000000E+00  0.000000E+00  0.000000E+00  0.000000E+00  0.000000E+00  0.000000E+00  0.000000E+00       Funtag_D
      77  6.088677E+05  5.714201E+05       37447.5       10.5972     0.0953349       1.45999      0.197027      0.230087     0.0105322   -0.00666249    0.00787308       1.02283       Opttag_D
      77       59.5292       2.13359       1.00000  0.000000E+00       1.00000  0.000000E+00      0.887985       54.9843       1.00000       19.1753  0.000000E+00       1.74000  0.000000E+00       1.74000  0.000000E+00  0.000000E+00      -3.48000       59.6917       1.81913        1       Scatag_D
      77       1742.64       5703.80  0.000000E+00  0.000000E+00       2525.40  0.000000E+00       2753.12       5419.33  0.000000E+00       10484.3       51.5211       8767.44 -1.000000E+10 -1.000000E+10 -1.000000E+10 -1.000000E+10 -1.000000E+10  0.000000E+00  0.000000E+00  0.000000E+00  0.000000E+00  0.000000E+00  0.000000E+00  0.000000E+00  0.000000E+00  0.000000E+00  0.000000E+00  0.000000E+00  0.000000E+00       Funtag_D

 An atom has moved more than     1.50 A from the initial/last
 position - if this was an accepted trial point, probably need to update the
 non-bonded contact list.


 An atom has moved more than     1.50 A from the initial/last
 position - if this was an accepted trial point, probably need to update the
 non-bonded contact list.

      78  6.088374E+05  5.714506E+05       37386.8       10.0435     0.0992168       1.59093      0.197064      0.230254     0.0104652   -0.00691092    0.00790757       1.02306       Opttag_D
      78       59.5310       2.13425       1.00000  0.000000E+00       1.00000  0.000000E+00      0.887942       55.0003       1.00000       19.2042  0.000000E+00       1.74111  0.000000E+00       1.74111  0.000000E+00  0.000000E+00      -3.48222       59.6917       1.81913        2       Scatag_D
      78       1757.94       5706.30  0.000000E+00  0.000000E+00       2527.94  0.000000E+00       2758.59       5421.83  0.000000E+00       10479.3       51.2654       8683.67 -1.000000E+10 -1.000000E+10 -1.000000E+10 -1.000000E+10 -1.000000E+10  0.000000E+00  0.000000E+00  0.000000E+00  0.000000E+00  0.000000E+00  0.000000E+00  0.000000E+00  0.000000E+00  0.000000E+00  0.000000E+00  0.000000E+00  0.000000E+00       Funtag_D

 An atom has moved more than     1.50 A from the initial/last
 position - need to update the non-bonded contact list.


 ############################################################################################################
 ###################################################### setting up contact terms
 ############################################################################################################

      79  6.088043E+05  5.715306E+05       37273.6       14.8369      0.105735       1.81289      0.197143      0.230529     0.0102884   -0.00738956    0.00800419       1.02371       Opttag_D
      79       59.5346       2.13622       1.00000  0.000000E+00       1.00000  0.000000E+00      0.887873       55.0258       1.00000       19.2583  0.000000E+00       1.74353  0.000000E+00       1.74353  0.000000E+00  0.000000E+00      -3.48707       59.6917       1.81913        1       Scatag_D
      79       1801.17       5713.65  0.000000E+00  0.000000E+00       2532.71  0.000000E+00       2765.31       5432.09  0.000000E+00       10479.7       50.9236       8498.10 -1.000000E+10 -1.000000E+10 -1.000000E+10 -1.000000E+10 -1.000000E+10  0.000000E+00  0.000000E+00  0.000000E+00  0.000000E+00  0.000000E+00  0.000000E+00  0.000000E+00  0.000000E+00  0.000000E+00  0.000000E+00  0.000000E+00  0.000000E+00       Funtag_D
      80  6.087510E+05  5.715025E+05       37248.5       25.4965      0.103516       1.75328      0.197117      0.230462     0.0103506   -0.00725872    0.00788750       1.02307       Opttag_D
      80       59.5333       2.13550       1.00000  0.000000E+00       1.00000  0.000000E+00      0.887885       55.0164       1.00000       19.2418  0.000000E+00       1.74268  0.000000E+00       1.74268  0.000000E+00  0.000000E+00      -3.48536       59.6917       1.81913        1       Scatag_D
      80       1749.03       5706.45  0.000000E+00  0.000000E+00       2531.45  0.000000E+00       2756.38       5464.82  0.000000E+00       10479.5       50.7230       8510.12 -1.000000E+10 -1.000000E+10 -1.000000E+10 -1.000000E+10 -1.000000E+10  0.000000E+00  0.000000E+00  0.000000E+00  0.000000E+00  0.000000E+00  0.000000E+00  0.000000E+00  0.000000E+00  0.000000E+00  0.000000E+00  0.000000E+00  0.000000E+00       Funtag_D
      81  6.086604E+05  5.715016E+05       37158.8       16.2606      0.104513       1.78939      0.197121      0.230498     0.0103525   -0.00729049    0.00787493       1.02309       Opttag_D
      81       59.5339       2.13531       1.00000  0.000000E+00       1.00000  0.000000E+00      0.887872       55.0176       1.00000       19.2518  0.000000E+00       1.74307  0.000000E+00       1.74307  0.000000E+00  0.000000E+00      -3.48614       59.6917       1.81913        1       Scatag_D
      81       1743.46       5706.71  0.000000E+00  0.000000E+00       2532.23  0.000000E+00       2756.01       5431.37  0.000000E+00       10480.2       51.1004       8457.73 -1.000000E+10 -1.000000E+10 -1.000000E+10 -1.000000E+10 -1.000000E+10  0.000000E+00  0.000000E+00  0.000000E+00  0.000000E+00  0.000000E+00  0.000000E+00  0.000000E+00  0.000000E+00  0.000000E+00  0.000000E+00  0.000000E+00  0.000000E+00       Funtag_D
      82  6.085866E+05  5.715329E+05       37053.7       18.8516      0.107883       1.90729      0.197153      0.230636     0.0102835   -0.00746997    0.00789920       1.02383       Opttag_D
      82       59.5361       2.13587       1.00000  0.000000E+00       1.00000  0.000000E+00      0.887837       55.0257       1.00000       19.2827  0.000000E+00       1.74455  0.000000E+00       1.74455  0.000000E+00  0.000000E+00      -3.48910       59.6917       1.81913        1       Scatag_D
      82       1754.22       5714.91  0.000000E+00  0.000000E+00       2534.90  0.000000E+00       2758.26       5437.35  0.000000E+00       10483.6       51.3901       8319.12 -1.000000E+10 -1.000000E+10 -1.000000E+10 -1.000000E+10 -1.000000E+10  0.000000E+00  0.000000E+00  0.000000E+00  0.000000E+00  0.000000E+00  0.000000E+00  0.000000E+00  0.000000E+00  0.000000E+00  0.000000E+00  0.000000E+00  0.000000E+00       Funtag_D
      83  6.084641E+05  5.715235E+05       36940.6       24.6491      0.109935       1.98179      0.197155      0.230667     0.0103043   -0.00739062    0.00788227       1.02433       Opttag_D
      83       59.5382       2.13514       1.00000  0.000000E+00       1.00000  0.000000E+00      0.887815       55.0221       1.00000       19.3035  0.000000E+00       1.74581  0.000000E+00       1.74581  0.000000E+00  0.000000E+00      -3.49161       59.6917       1.81913        1       Scatag_D
      83       1746.71       5720.48  0.000000E+00  0.000000E+00       2536.59  0.000000E+00       2758.65       5458.63  0.000000E+00       10488.2       52.0905       8179.23 -1.000000E+10 -1.000000E+10 -1.000000E+10 -1.000000E+10 -1.000000E+10  0.000000E+00  0.000000E+00  0.000000E+00  0.000000E+00  0.000000E+00  0.000000E+00  0.000000E+00  0.000000E+00  0.000000E+00  0.000000E+00  0.000000E+00  0.000000E+00       Funtag_D
      84  6.083404E+05  5.714923E+05       36848.1       19.7502      0.110928       2.01714      0.197139      0.230640     0.0103730   -0.00722605    0.00788606       1.02498       Opttag_D
      84       59.5399       2.13383       1.00000  0.000000E+00       1.00000  0.000000E+00      0.887808       55.0128       1.00000       19.3139  0.000000E+00       1.74649  0.000000E+00       1.74649  0.000000E+00  0.000000E+00      -3.49298       59.6917       1.81913        1       Scatag_D
      84       1748.39       5727.81  0.000000E+00  0.000000E+00       2537.48  0.000000E+00       2760.17       5444.83  0.000000E+00       10492.2       52.0729       8085.14 -1.000000E+10 -1.000000E+10 -1.000000E+10 -1.000000E+10 -1.000000E+10  0.000000E+00  0.000000E+00  0.000000E+00  0.000000E+00  0.000000E+00  0.000000E+00  0.000000E+00  0.000000E+00  0.000000E+00  0.000000E+00  0.000000E+00  0.000000E+00       Funtag_D
      85  6.083045E+05  5.714095E+05       36895.0       20.9301      0.107499       1.90080      0.197081      0.230433     0.0105557   -0.00674846    0.00789981       1.02558       Opttag_D
      85       59.5395       2.13156       1.00000  0.000000E+00       1.00000  0.000000E+00      0.887836       54.9920       1.00000       19.2847  0.000000E+00       1.74538  0.000000E+00       1.74538  0.000000E+00  0.000000E+00      -3.49076       59.6917       1.81913        1       Scatag_D
      85       1754.50       5734.47  0.000000E+00  0.000000E+00       2534.72  0.000000E+00       2758.97       5450.48  0.000000E+00       10499.7       52.0950       8110.06 -1.000000E+10 -1.000000E+10 -1.000000E+10 -1.000000E+10 -1.000000E+10  0.000000E+00  0.000000E+00  0.000000E+00  0.000000E+00  0.000000E+00  0.000000E+00  0.000000E+00  0.000000E+00  0.000000E+00  0.000000E+00  0.000000E+00  0.000000E+00       Funtag_D
      86  6.082046E+05  5.714344E+05       36770.2       18.4161      0.110571       2.00718      0.197110      0.230558     0.0105009   -0.00699554    0.00786077       1.02528       Opttag_D
      86       59.5410       2.13195       1.00000  0.000000E+00       1.00000  0.000000E+00      0.887805       54.9952       1.00000       19.3117  0.000000E+00       1.74614  0.000000E+00       1.74614  0.000000E+00  0.000000E+00      -3.49229       59.6917       1.81913        1       Scatag_D
      86       1737.20       5731.15  0.000000E+00  0.000000E+00       2537.60  0.000000E+00       2761.31       5445.34  0.000000E+00       10497.7       51.9006       8007.95 -1.000000E+10 -1.000000E+10 -1.000000E+10 -1.000000E+10 -1.000000E+10  0.000000E+00  0.000000E+00  0.000000E+00  0.000000E+00  0.000000E+00  0.000000E+00  0.000000E+00  0.000000E+00  0.000000E+00  0.000000E+00  0.000000E+00  0.000000E+00       Funtag_D
      87  6.081550E+05  5.714329E+05       36722.1       20.8142      0.110213       1.99246      0.197107      0.230576     0.0105042   -0.00708753    0.00783697       1.02415       Opttag_D
      87       59.5396       2.13197       1.00000  0.000000E+00       1.00000  0.000000E+00      0.887806       54.9955       1.00000       19.3074  0.000000E+00       1.74544  0.000000E+00       1.74544  0.000000E+00  0.000000E+00      -3.49088       59.6917       1.81913        1       Scatag_D
      87       1726.69       5718.50  0.000000E+00  0.000000E+00       2536.99  0.000000E+00       2759.58       5454.50  0.000000E+00       10491.4       51.5745       7982.88 -1.000000E+10 -1.000000E+10 -1.000000E+10 -1.000000E+10 -1.000000E+10  0.000000E+00  0.000000E+00  0.000000E+00  0.000000E+00  0.000000E+00  0.000000E+00  0.000000E+00  0.000000E+00  0.000000E+00  0.000000E+00  0.000000E+00  0.000000E+00       Funtag_D
      88  6.081045E+05  5.714200E+05       36684.5       9.57618      0.110023       1.98819      0.197101      0.230550     0.0105325   -0.00702610    0.00783614       1.02418       Opttag_D
      88       59.5396       2.13157       1.00000  0.000000E+00       1.00000  0.000000E+00      0.887806       54.9917       1.00000       19.3059  0.000000E+00       1.74537  0.000000E+00       1.74537  0.000000E+00  0.000000E+00      -3.49074       59.6917       1.81913        1       Scatag_D
      88       1726.33       5718.79  0.000000E+00  0.000000E+00       2536.90  0.000000E+00       2759.61       5427.54  0.000000E+00       10492.8       51.6498       7970.83 -1.000000E+10 -1.000000E+10 -1.000000E+10 -1.000000E+10 -1.000000E+10  0.000000E+00  0.000000E+00  0.000000E+00  0.000000E+00  0.000000E+00  0.000000E+00  0.000000E+00  0.000000E+00  0.000000E+00  0.000000E+00  0.000000E+00  0.000000E+00       Funtag_D
      89  6.080630E+05  5.713993E+05       36663.7       8.08553      0.109850       1.98631      0.197091      0.230515     0.0105784   -0.00694781    0.00783850       1.02438       Opttag_D
      89       59.5392       2.13090       1.00000  0.000000E+00       1.00000  0.000000E+00      0.887805       54.9850       1.00000       19.3042  0.000000E+00       1.74515  0.000000E+00       1.74515  0.000000E+00  0.000000E+00      -3.49030       59.6917       1.81913        1       Scatag_D
      89       1727.37       5721.08  0.000000E+00  0.000000E+00       2536.81  0.000000E+00       2759.90       5429.40  0.000000E+00       10493.8       51.6837       7943.70 -1.000000E+10 -1.000000E+10 -1.000000E+10 -1.000000E+10 -1.000000E+10  0.000000E+00  0.000000E+00  0.000000E+00  0.000000E+00  0.000000E+00  0.000000E+00  0.000000E+00  0.000000E+00  0.000000E+00  0.000000E+00  0.000000E+00  0.000000E+00       Funtag_D
      90  6.080221E+05  5.713826E+05       36639.4       9.71411      0.109648       1.98621      0.197082      0.230481     0.0106151   -0.00687749    0.00784078       1.02440       Opttag_D
      90       59.5383       2.13035       1.00000  0.000000E+00       1.00000  0.000000E+00      0.887804       54.9796       1.00000       19.3017  0.000000E+00       1.74494  0.000000E+00       1.74494  0.000000E+00  0.000000E+00      -3.48988       59.6917       1.81913        1       Scatag_D
      90       1728.37       5721.31  0.000000E+00  0.000000E+00       2536.48  0.000000E+00       2759.68       5435.54  0.000000E+00       10492.3       51.7113       7914.04 -1.000000E+10 -1.000000E+10 -1.000000E+10 -1.000000E+10 -1.000000E+10  0.000000E+00  0.000000E+00  0.000000E+00  0.000000E+00  0.000000E+00  0.000000E+00  0.000000E+00  0.000000E+00  0.000000E+00  0.000000E+00  0.000000E+00  0.000000E+00       Funtag_D
      91  6.079741E+05  5.713692E+05       36604.9       15.4868      0.109525       2.00282      0.197071      0.230407     0.0106448   -0.00669667    0.00784187       1.02432       Opttag_D
      91       59.5366       2.12992       1.00000  0.000000E+00       1.00000  0.000000E+00      0.887801       54.9751       1.00000       19.2988  0.000000E+00       1.74490  0.000000E+00       1.74490  0.000000E+00  0.000000E+00      -3.48979       59.6917       1.81913        1       Scatag_D
      91       1728.85       5720.41  0.000000E+00  0.000000E+00       2535.81  0.000000E+00       2758.89       5447.34  0.000000E+00       10489.1       52.0420       7872.55 -1.000000E+10 -1.000000E+10 -1.000000E+10 -1.000000E+10 -1.000000E+10  0.000000E+00  0.000000E+00  0.000000E+00  0.000000E+00  0.000000E+00  0.000000E+00  0.000000E+00  0.000000E+00  0.000000E+00  0.000000E+00  0.000000E+00  0.000000E+00       Funtag_D
      92  6.079388E+05  5.713712E+05       36567.6       10.8475      0.109453       2.01714      0.197071      0.230345     0.0106404   -0.00657426    0.00784375       1.02414       Opttag_D
      92       59.5355       2.13003       1.00000  0.000000E+00       1.00000  0.000000E+00      0.887799       54.9767       1.00000       19.2965  0.000000E+00       1.74516  0.000000E+00       1.74516  0.000000E+00  0.000000E+00      -3.49031       59.6917       1.81913        1       Scatag_D
      92       1729.68       5718.36  0.000000E+00  0.000000E+00       2535.20  0.000000E+00       2759.08       5431.26  0.000000E+00       10486.8       52.1607       7855.04 -1.000000E+10 -1.000000E+10 -1.000000E+10 -1.000000E+10 -1.000000E+10  0.000000E+00  0.000000E+00  0.000000E+00  0.000000E+00  0.000000E+00  0.000000E+00  0.000000E+00  0.000000E+00  0.000000E+00  0.000000E+00  0.000000E+00  0.000000E+00       Funtag_D
      93  6.079127E+05  5.713787E+05       36534.0       6.33736      0.109550       2.02007      0.197074      0.230351     0.0106237   -0.00657546    0.00783490       1.02342       Opttag_D
      93       59.5359       2.13031       1.00000  0.000000E+00       1.00000  0.000000E+00      0.887798       54.9807       1.00000       19.2975  0.000000E+00       1.74521  0.000000E+00       1.74521  0.000000E+00  0.000000E+00      -3.49042       59.6917       1.81913        1       Scatag_D
      93       1725.78       5710.31  0.000000E+00  0.000000E+00       2535.09  0.000000E+00       2758.11       5421.33  0.000000E+00       10485.6       52.0532       7845.68 -1.000000E+10 -1.000000E+10 -1.000000E+10 -1.000000E+10 -1.000000E+10  0.000000E+00  0.000000E+00  0.000000E+00  0.000000E+00  0.000000E+00  0.000000E+00  0.000000E+00  0.000000E+00  0.000000E+00  0.000000E+00  0.000000E+00  0.000000E+00       Funtag_D
      94  6.078838E+05  5.713872E+05       36496.5       6.26299      0.109645       2.02875      0.197079      0.230328     0.0106049   -0.00651313    0.00782766       1.02275       Opttag_D
      94       59.5363       2.13071       1.00000  0.000000E+00       1.00000  0.000000E+00      0.887795       54.9868       1.00000       19.2981  0.000000E+00       1.74525  0.000000E+00       1.74525  0.000000E+00  0.000000E+00      -3.49050       59.6917       1.81913        1       Scatag_D
      94       1722.59       5702.87  0.000000E+00  0.000000E+00       2534.69  0.000000E+00       2757.02       5413.48  0.000000E+00       10484.7       51.8542       7829.31 -1.000000E+10 -1.000000E+10 -1.000000E+10 -1.000000E+10 -1.000000E+10  0.000000E+00  0.000000E+00  0.000000E+00  0.000000E+00  0.000000E+00  0.000000E+00  0.000000E+00  0.000000E+00  0.000000E+00  0.000000E+00  0.000000E+00  0.000000E+00       Funtag_D
      95  6.078587E+05  5.713920E+05       36466.6       10.4193      0.109725       2.04058      0.197081      0.230270     0.0105944   -0.00637672    0.00782729       1.02247       Opttag_D
      95       59.5369       2.13108       1.00000  0.000000E+00       1.00000  0.000000E+00      0.887789       54.9934       1.00000       19.2986  0.000000E+00       1.74513  0.000000E+00       1.74513  0.000000E+00  0.000000E+00      -3.49027       59.6917       1.81913        1       Scatag_D
      95       1722.43       5699.73  0.000000E+00  0.000000E+00       2533.88  0.000000E+00       2755.96       5408.75  0.000000E+00       10486.5       51.5369       7807.89 -1.000000E+10 -1.000000E+10 -1.000000E+10 -1.000000E+10 -1.000000E+10  0.000000E+00  0.000000E+00  0.000000E+00  0.000000E+00  0.000000E+00  0.000000E+00  0.000000E+00  0.000000E+00  0.000000E+00  0.000000E+00  0.000000E+00  0.000000E+00       Funtag_D
      96  6.078412E+05  5.713852E+05       36456.0       7.63024      0.109734       2.04084      0.197079      0.230246     0.0106095   -0.00632296    0.00782552       1.02243       Opttag_D
      96       59.5375       2.13094       1.00000  0.000000E+00       1.00000  0.000000E+00      0.887783       54.9949       1.00000       19.2993  0.000000E+00       1.74494  0.000000E+00       1.74494  0.000000E+00  0.000000E+00      -3.48988       59.6917       1.81913        1       Scatag_D
      96       1721.65       5699.37  0.000000E+00  0.000000E+00       2533.64  0.000000E+00       2755.25       5404.73  0.000000E+00       10488.5       51.3875       7801.46 -1.000000E+10 -1.000000E+10 -1.000000E+10 -1.000000E+10 -1.000000E+10  0.000000E+00  0.000000E+00  0.000000E+00  0.000000E+00  0.000000E+00  0.000000E+00  0.000000E+00  0.000000E+00  0.000000E+00  0.000000E+00  0.000000E+00  0.000000E+00       Funtag_D
      97  6.078287E+05  5.713752E+05       36453.5       4.51439      0.109681       2.03457      0.197075      0.230255     0.0106316   -0.00634675    0.00782523       1.02246       Opttag_D
      97       59.5376       2.13056       1.00000  0.000000E+00       1.00000  0.000000E+00      0.887784       54.9914       1.00000       19.2994  0.000000E+00       1.74480  0.000000E+00       1.74480  0.000000E+00  0.000000E+00      -3.48961       59.6917       1.81913        1       Scatag_D
      97       1721.52       5699.61  0.000000E+00  0.000000E+00       2533.76  0.000000E+00       2755.15       5406.32  0.000000E+00       10488.2       51.4406       7797.54 -1.000000E+10 -1.000000E+10 -1.000000E+10 -1.000000E+10 -1.000000E+10  0.000000E+00  0.000000E+00  0.000000E+00  0.000000E+00  0.000000E+00  0.000000E+00  0.000000E+00  0.000000E+00  0.000000E+00  0.000000E+00  0.000000E+00  0.000000E+00       Funtag_D
      98  6.078180E+05  5.713575E+05       36460.5       3.85450      0.109616       2.02985      0.197064      0.230246     0.0106705   -0.00631980    0.00782809       1.02267       Opttag_D
      98       59.5378       2.12994       1.00000  0.000000E+00       1.00000  0.000000E+00      0.887782       54.9868       1.00000       19.2998  0.000000E+00       1.74453  0.000000E+00       1.74453  0.000000E+00  0.000000E+00      -3.48907       59.6917       1.81913        1       Scatag_D
      98       1722.78       5701.97  0.000000E+00  0.000000E+00       2533.74  0.000000E+00       2755.02       5411.15  0.000000E+00       10489.2       51.5310       7795.12 -1.000000E+10 -1.000000E+10 -1.000000E+10 -1.000000E+10 -1.000000E+10  0.000000E+00  0.000000E+00  0.000000E+00  0.000000E+00  0.000000E+00  0.000000E+00  0.000000E+00  0.000000E+00  0.000000E+00  0.000000E+00  0.000000E+00  0.000000E+00       Funtag_D

   MaxLik Scaling Cycle 0105

   Wilson plot

   Statistics in resolution bins after refinement cycle 0003
```


|  |  |
| --- | --- |
|  | - Rxpct factors plot- Rxpct factors table- <Log-Lik Gain>- Ln Sigma A- Correlation Coefficients |


```
   Iters     Tot_Funct    Xray_Funct    Geom_Funct          Grms     rmsD2init     maxD2init             R         Rfree           LLG       LLGfree     rms(bond)    rms(angle)
   Iters             K   B_IMPF_FRAG        K_MISS   B_TEMP_MISS   K_IMPF_MISS   B_IMPF_MISS        K_SOLV   B_TEMP_SOLV   K_IMPF_SOLV   B_IMPF_SOLV        B_TEMP     B_TEMP_11     B_TEMP_12     B_TEMP_22     B_TEMP_13     B_TEMP_23     B_TEMP_33      K_WILSON      B_WILSON      FGCYCLES
   Iters          BOND         ANGLE      IMPROPER       TORSION    SINTORSION      TRIGONAL        PLANAR  BCORRELATION       CONTACT     IDEALDIST        CHIRAL    SIMILARITY       OCCSSUM  UTILDISTANCE     UTILANGLE   UTILTORSION        RHOFIT        ADP-01        ADP-02        ADP-03        ADP-04        ADP-05        ADP-06        ADP-07        ADP-08        ADP-09        ADP-10      RHOFITPC            QM
      99  6.078111E+05  5.713431E+05       36468.0       5.87844      0.109581       2.03164      0.197057      0.230226     0.0107022   -0.00626921    0.00783058       1.02287       Opttag_D
      99       59.5381       2.12945       1.00000  0.000000E+00       1.00000  0.000000E+00      0.887781       54.9839       1.00000       19.3001  0.000000E+00       1.74434  0.000000E+00       1.74434  0.000000E+00  0.000000E+00      -3.48867       59.6917       1.81913        1       Scatag_D
      99       1723.88       5704.28  0.000000E+00  0.000000E+00       2533.67  0.000000E+00       2754.79       5414.11  0.000000E+00       10490.5       51.6114       7795.14 -1.000000E+10 -1.000000E+10 -1.000000E+10 -1.000000E+10 -1.000000E+10  0.000000E+00  0.000000E+00  0.000000E+00  0.000000E+00  0.000000E+00  0.000000E+00  0.000000E+00  0.000000E+00  0.000000E+00  0.000000E+00  0.000000E+00  0.000000E+00       Funtag_D
   Geometry Restraint Deviations SCREEN Information

 
 No -bcorrel_zeroocchydrogens_factor flag found - keeping default: will set B for OCC=0 hydrogens to    1.200 times heavy-atom B


 TLS information. Total number of groups:     2


 Group   Natoms    FixRB   FixTLS   Group Tag    Group Spec
     1     2770      Y        Y     tls1    { A|* }
     2     2774      Y        Y     tls2    { B|* }
 
NOTE BUSTER_TLS_SET tls1 { A|* }
NOTE BUSTER_TLS_T   tls1     -2.09805E-01 -1.15711E-01  3.73362E-01 -2.40920E-02  4.96139E-02  2.21940E-02
NOTE BUSTER_TLS_L   tls1      1.24514E+00  1.05226E+00  6.66191E-01  1.62361E-01 -3.88521E-01  3.54587E-01
NOTE BUSTER_TLS_S   tls1      2.06506E-01 -1.53727E-01  1.93700E-01 -1.05534E-01 -6.67227E-02 -9.37109E-02 -4.76199E-02 -4.26923E-02
NOTE BUSTER_TLS_O   tls1     -2.33748E+01 -8.36624E+01  1.14308E+01
NOTE BUSTER_TLS_FIX tls1 ALL
 
NOTE BUSTER_TLS_SET tls2 { B|* }
NOTE BUSTER_TLS_T   tls2     -2.19975E-01 -1.58351E-01  4.94077E-01  1.93068E-02  4.62273E-02  2.44113E-03
NOTE BUSTER_TLS_L   tls2      1.30038E+00  8.42752E-01  9.70856E-01  6.65317E-01 -8.38129E-01 -5.84650E-01
NOTE BUSTER_TLS_S   tls2     -2.69202E-02  1.09150E-01 -8.14403E-02  6.77598E-02  1.92600E-01  1.97964E-02 -6.29584E-02  2.14661E-02
NOTE BUSTER_TLS_O   tls2      2.20570E+01 -7.19562E+01  3.49479E+00
NOTE BUSTER_TLS_FIX tls2 ALL

 For final position, Geometry function summary
     term                           number     weight       rms        GooF      function     fn/numb
     ================================================================================================
     BOND bond lengths (angs)       5660       2.000      0.0078       0.390      1723.88       0.305
     ANGL bond angles (degs)        7681       2.000        1.02       0.609      5704.28       0.743
     TORS split-harmonic tors       1270       0.000       18.21       1.146         0.00       0.000
     SINTOR sinusoidal tors         1270       2.000       18.21                  2533.67       1.995
     PLAN general plane (angs)       964       5.000      0.0151       0.756      2754.79       2.858
     BCORrelations (angs^2)         5660      10.000       0.867       0.309      5414.11       0.957
     CONT (bad contacts)               0       5.000                                 0.00
     IDEAL(ideal-dist contact)      5091       4.000                             10490.55
     CHIRAL (gelly semiharmon)       740       5.000                                51.61
     SIM similarity restraint for NCS and/or TARGET                               7795.14
     OCCSUM restraint on sum of occupanies                                           0.00       as none defined
     DISTAN utility restraints interatomic distance                                  0.00       as none defined
     UTILANGLE utility angle restraints                                              0.00       as none defined
     UTILTOR utility restraints on torsion angles                                    0.00       as none defined
     Peptide omega torsion angles                           3.12 degs - monitor only

 Weighted rms      0.0078         1.023  (temporary rms bond and angle for BUSTER to pick up!)
 Weighted intra-residual rms(bond) for StdProtein (excluding H)      0.0076
 Weighted intra-residual rms(bond) for Buffer     (excluding H)      0.0033
 Outputing geometry progress graph in plotmtv format to file:Geom_Res.mtv

 ############### Running final geometry/screen analysis (screen_final.txt)

 s/r gelly normal termination
```

Statistics and scale factors vs. cycle number
```

|  |  |  |
| --- | --- | --- |
|  | - Rxpct factors- <Log-Lik Gain>- GEOM Residuals- Ln Sigma_A_ | - Scale Factors- B Factors- Imperfection Factors |

```
 Writing F_calc(model+solvent) to complete.mtz

   Final Geometry Information


---


### 2. Final results

Statistics and scale factors vs. cycle number
```

|  |  |  |
| --- | --- | --- |
|  | - Rxpct factors- <Log-Lik Gain>- GEOM Residuals- Ln Sigma_A_ | - Scale Factors- B Factors- Imperfection Factors |

```
   Average Figure of Merit
   Structure Factor Amplitudes


 # normal termination

  Program stopped.
```

closesok message: time spent in tree = 1580.80 (user) 36.91 (sys)

---

# Normal termination

```
 Resources usage summary:
     Time spent in client ...................  1580.81 (user)       36.94 (system)
     Time spent in scripts (    27 calls) ...     2.74 (user)        0.16 (system)
     Time spent in server ...................     0.00 (user)        0.00 (system)

     Max resident memory used (kb) .......... 449480
     Number of page faults .................. 0 (major)    1170659 (minor)
     Number of context switches ............. 21810 (niced)    6066 (other)
```
